# A Systematic Review and Meta-Analysis of the Effects of Flavanol-Containing Tea, Cocoa and Apple Products on Body Composition and Blood Lipids: Exploring the Factors Responsible for Variability in Their Efficacy

**DOI:** 10.3390/nu9070746

**Published:** 2017-07-13

**Authors:** Antonio González-Sarrías, Emilie Combet, Paula Pinto, Pedro Mena, Margherita Dall’Asta, Mar Garcia-Aloy, Ana Rodríguez-Mateos, Eileen R. Gibney, Julie Dumont, Marika Massaro, Julio Sánchez-Meca, Christine Morand, María-Teresa García-Conesa

**Affiliations:** 1 Research Group on Quality, Safety and Bioactivity of Plant Foods, Campus de Espinardo, Centro de Edafologia y Biologia Aplicada del Segura-Consejo Superior de Investigaciones Científicas (CEBAS-CSIC), P.O. Box 164, 30100 Murcia, Spain; 2 Human Nutrition, School of Medicine, Dentistry and Nursing, College of Medical, Veterinary and Life Sciences, University of Glasgow, Glasgow G31 2ER, UK; Emilie.CombetAspray@glasgow.ac.uk; 3 Polytechnic Institute of Santarem, Escola Superior Agrária (ESA), Department of Food Technology, Biotechnology and Nutrition, 2001-904 Santarém, Portugal; paula.pinto@esa.ipsantarem.pt; 4 Human Nutrition Unit, Department of Food & Drug, University of Parma, 43125 Parma, Italy; pedromiguel.menaparreno@unipr.it (P.M.); margherita.dallasta@unipr.it (M.D.); 5 Biomarkers and Nutrimetabolomic Laboratory, Department of Nutrition, Food Sciences and Gastronomy, University of Barcelona, 08028 Barcelona, Spain; margarcia@ub.edu; 6 CIBER de Fragilidad y Envejecimiento Saludable (CIBERFES), Instituto de Salud Carlos III, 08028 Barcelona, Spain; 7 Division of Diabetes and Nutritional Sciences, King’s College London, London SE1 9NH, UK; ana.rodriguez-mateos@kcl.ac.uk; 8 Institute of Food and Health, School of Agriculture and Food Science, University College Dublin (UCD), Belfield, Dublin 4, Ireland; eileen.gibney@ucd.ie; 9 U1167-RID-AGE-Facteurs de risque et Déterminants Moléculaires des Maladies Liées au Vieillissement, University Lille, Institut National de la Santé et de la Recherche Médicale (INSERM), Centre Hospitalier Universitaire (CHU) Lille, Institut Pasteur de Lille, F-59000 Lille, France; julie.dumont@pasteur-lille.fr; 10 National Research Council (CNR), Institute of Clinical Physiology, 73100 Lecce, Italy; marika@ifc.cnr.it; 11 Department of Basic Psychology & Methodology, Faculty of Psychology, University of Murcia, 30100 Murcia, Spain; jsmeca@um.es; 12 Institut National de la Recherche Agronomique (INRA), Human Nutrition Unit, Université Clermont Auvergne (UCA), Centre de Recherches en Nutrition Humaine (CRNH) Auvergne, F-63000 Clermont-Ferrand, France; christine.morand@inra.fr

**Keywords:** flavanols, tea, cocoa, apple, cardiometabolic disorders, meta-analysis, interindividual variability, blood lipids, body mass index, waist circumference

## Abstract

Several randomized controlled trials (RCTs) and meta-analyses support the benefits of flavanols on cardiometabolic health, but the factors affecting variability in the responses to these compounds have not been properly assessed. The objectives of this meta-analysis were to systematically collect the RCTs-based-evidence of the effects of flavanol-containing tea, cocoa and apple products on selected biomarkers of cardiometabolic risk and to explore the influence of various factors on the variability in the responses to the consumption of these products. A total of 120 RCTs were selected. Despite a high heterogeneity, the intake of the flavanol-containing products was associated using a random model with changes (reported as standardized difference in means (SDM)) in body mass index (−0.15, *p* < 0.001), waist circumference (−0.29, *p* < 0.001), total-cholesterol (−0.21, *p* < 0.001), LDL-cholesterol (−0.23, *p* < 0.001), and triacylglycerides (−0.11, *p* = 0.027), and with an increase of HDL-cholesterol (0.15, *p* = 0.005). Through subgroup analyses, we showed the influence of baseline-BMI, sex, source/form of administration, medication and country of investigation on some of the outcome measures and suggest that flavanols may be more effective in specific subgroups such as those with a BMI ≥ 25.0 kg/m^2^, non-medicated individuals or by specifically using tea products. This meta-analysis provides the first robust evidence of the effects induced by the consumption of flavanol-containing tea, cocoa and apple products on weight and lipid biomarkers and shows the influence of various factors that can affect their bioefficacy in humans. Of note, some of these effects are quantitatively comparable to those produced by drugs, life-style changes or other natural products. Further, RCTs in well-characterized populations are required to fully comprehend the factors affecting inter-individual responses to flavanol and thereby improve flavanols efficacy in the prevention of cardiometabolic disorders.

## 1. Introduction

Metabolic disorders, principally, abdominal obesity, dyslipidemia (high levels of triacylglycerides (TAGs) and low levels of high-density lipoprotein (HDL)), and insulin resistance have been associated to an increased risk of Type-2 diabetes mellitus (DM) and cardiovascular diseases (CVDs). CVDs remain the number one cause of death in developed countries and their prevalence is increasing rapidly in developing nations and in adolescents [1]. It is now well established from population studies that some aspects of CVDs risk can be modulated by various dietary interventions including an increased consumption of plant foods [2], as part of a healthy balanced diet. In addition to other protective compounds (i.e., fiber and vitamins), plant foods are an exclusive and abundant source of phytochemicals, a large and diverse group of compounds which exhibit an array of biological activities. The intake of these bioactive compounds are thought to contribute to the health benefits associated with the consumption of such foods [3]. Polyphenols are some of the most abundant phytochemicals in plant foods and increasing evidence from cohort studies indicate that the intake of some of these compounds such as diverse flavonoids and/or, importantly, some of their derived microbial metabolites (e.g., enterolactone) may help to reduce the development of CVDs and CVDs mortality risk [4,5,6,7]. This evidence is supported by animal and clinical studies reporting beneficial effects of the consumption of some polyphenol-rich foods or pure compounds on CVDs risk factors such as blood cholesterol, blood pressure, endothelial function and arterial stiffness [8].

Polyphenols encompass several families of compounds, the most represented in plant foods being phenolic acids and flavonoids [9]. A major group of flavonoids is constituted by flavanols that are abundant in green tea, red wine, cocoa and various fruits such as apples [10]. A summary of the major flavonoids present in tea (green and black), cocoa powder and apple is shown in Table S1. The assessment of daily intakes of flavanols across Europe revealed a large variation between countries (from 200 to 800 mg/day) depending on their dietary habits and the intake of tea [11]. The flavanol group is composed primarily of the epicatechin and catechin monomers, and of their oligomeric and polymeric forms, the procyanidins. Flavanol monomers and dimeric procyanidins are bioavailable. They undergo extensive phase II conjugation and are found in the blood circulation mostly as *O*-methylated, sulfated and glucuronidated conjugates (nM to μM range) [12,13]. In contrast, the procyanidins polymers are not absorbed and do not contribute to the systemic pool of flavanols in humans [14].

Human studies remain essential to understanding the effects of the plant bioactive compounds on health and thus, an increasing number of randomized controlled trials (RCTs) with flavanol-containing products have been carried out over the past two decades. Meta-analyses constitute a useful tool to integrate the accumulated RCTs and review the evidence in humans. Some of the main problems affecting the results of meta-analyses are the usually limited number of studies included as well as a range of factors that introduce heterogeneity in the findings. Identifying the factors underlying variability, as well as developing new and innovative methodologies to account for such variability constitute an overarching goal to ultimately optimize the beneficial health effects of plant food bioactives. Among the potential factors involved in such heterogeneity are: (i) factors inherent to the individuals: (epi) genetic factors, gut microbiota, baseline conditions (BMI, medication), sex, health status, ethnicity, and age; and (ii) factors intrinsic to the type of study (design, duration, dose, and type of product) [15].

The main goals of the present study were: (i) to systematically review and appraise, through meta-analysis, the impact of flavanol-containing tea, cocoa and apple products, three main sources of flavanols, on selected biomarkers of cardiometabolic risk, i.e., BMI, WC and blood lipid levels (total-, LDL-, HDL-cholesterol and TAGs); and (ii) to further explore some of the factors that may be implicated in the inter-individual variability in the response to the consumption of these flavanol-containing products.

## 2. Materials and Methods 

This systematic review and meta-analysis followed the PRISMA (Preferred Reporting Items for Systematic Reviews and Meta-Analyses) statement guidelines [16], the Cochrane Handbook for Systematic Reviews of Interventions [17], and the Centre for Reviews and Dissemination’s guidance for undertaking reviews in health care [18]. The protocol for this review was registered in the International Prospective Register of Systematic Reviews (PROSPERO, www.crd.york.ac.uk/prospero/index.asp) with the registration number CRD42016033878.

### 2.1. Search Strategy

A comprehensive search on PubMed and Web of Science databases was conducted in July 2015. Search terms included a combination of keywords referring to: (1) bioactive (polyphenols, flavonoids, flavanols, flavan-3-ol, (epi)catechin, (epi)gallocatechin gallate, theaflavins, thearubigin, and procyanidin); (2) food source (apple, tea, and cocoa); (3) type of study and participants (trial, experiment, study, intervention; human, subjects, men, women, patients, volunteers, and participants); and (4) cardiometabolic outcomes (BMI, WC, total cholesterol, LDL cholesterol, HDL cholesterol, and TAGs). No type of restriction was applied during the electronic searches. 

### 2.2. Study Selection and Data Extraction

Two authors independently assessed all papers and in the case of disagreement, discussed findings to reach a consensus, or in the absence of resolution, a third author was contacted. Studies included in the meta-analysis were limited to human RCTs testing the effect of flavanol-containing tea, cocoa or apple products, which had a control group receiving a placebo and measured one or more of the defined outcomes (BMI, WC, total cholesterol, LDL cholesterol, HDL cholesterol, or TAGs). Manuscripts written in any European language were included, whereas other manuscripts were excluded. Additionally, the studies with the following characteristics were excluded: studies with flavanol-rich food sources other than tea, cocoa or apples; and studies with multifactorial interventions (i.e., flavanols given as a part of a multicomponent treatment; dietary or physical activity co-intervention). Data extraction was performed in duplicate by two authors, independently, and cross-checked by a third author using a standardized data extraction form. Extracted data included publication details (year of publication, contact details, clinical trial and registration number); participant characteristics (geographical origin, total number of participants included in the study and in the analysis, sex distribution, age, ethnicity, health status, menopausal status, smoking habits, and use of medication); study setting and design (cross-over or parallel design, duration of the intervention, number of arms and description, number of participants located in each arm and completing the study, composition of test and placebo, and dose and mode of administration); and outcomes (type of sample, changes in the outcome, values before and after intervention, and *p*-value).

### 2.3. Assessment of Quality and Data Analysis

The quality of the studies was assessed based on the Cochrane Collaboration measurement with some modifications [19]. The specific items used for the assessments are detailed in a previous meta-analysis following the same protocol [7].

Data for each outcome were analyzed using the Comprehensive Meta-Analysis Software, version 3.0 (Biostat, Englewood, NJ, USA) [20]. The free scale index standardized difference in means (SDM) was used to combine data from the highest number of collected valid studies, increasing the pool of studies and the power to detect significant differences. SDM, standard error (SE) and the corresponding 95% confidence intervals (CI) were calculated and pooled using random effects models to determine test/placebo differences across studies. Statistical heterogeneity between studies was assessed by using the Cochran *Q* test, the between-studies variance (*T*^2^) and *I*^2^ (an estimate of the proportion of variance across studies caused by heterogeneity rather than by random errors) where *I*^2^ values equal to 25%, 50% and 75% were considered as low, moderate and high heterogeneity, respectively. Publication bias was assessed visually with funnel plots and statistically by applying the Egger’s regression test. Further assessment of the possible associations between the overall changes attributed to the supplementation with the flavanols and the duration of the intervention was examined using random-effects meta-regression analysis. Using the random model, we have additionally estimated the overall effect size as the difference in means (DM) and 95% CI.

Subgroup analyses were conducted to explore potential factors that may introduce heterogeneity into the studies and influence the inter-individual variability in the response to supplementation with the flavanol-containing products. We selected those factors that were more clearly described throughout articles (Table 1). We included factors that might be attributed to some of the individuals’ characteristics, such as baseline BMI, sex, smoking habits and medication/health status. Age or ethnicity could not be assessed due to unclear reporting. We also included stratification by the country in which the study was carried out, the source and form of administration of the flavanols, as well as the type of diet reported to be followed during the intervention. For each subgroup, the pooled effects (SDM) and the significance of this value were estimated. Additionally, statistical comparisons between subgroups were performed by applying a random-effects analysis and calculation of the between-categories Q statistic, the *p*-value and the R^2^ index (proportion of between-studies variance explained by each factor or covariate). Using some of the factors that partially explained some of the between-studies variance for a particular variable, we applied a multiple meta-regression analysis with a random-effects model to search for a potential combination of factors that best explained the between-study variance for this variable. Statistical significance of the findings was as follows: *p*-value < 0.05 was considered significant, while *p*-value ≥ 0.05 and < 0.1 was considered marginally significant.

## 3. Results

### 3.1. Description of the Included Studies

**Figure 1:**
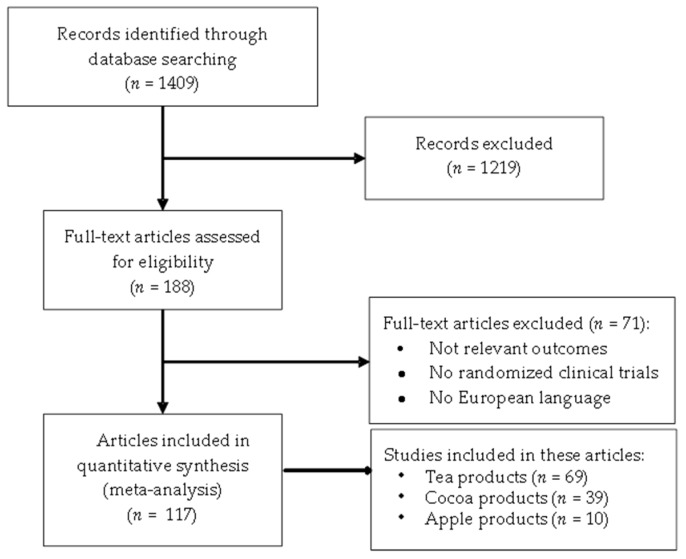
Flow diagram showing the study selection process.

A total of 1409 articles were initially identified through the search on the electronic databases. After removal of duplicates and screening, 188 trials were selected for data extraction. After detailed analysis of the full text, 71 articles were excluded, due to lack of relevant outcomes, aspects of study design or publication language. The final number of articles selected for meta-analysis was selected from a total of 117 articles published between 1997 and July 2015 (included) [21,22,23,24,25,26,27,28,29,30,31,32,33,34,35,36,37,38,39,40,41,42,43,44,45,46,47,48,49,50,51,52,53,54,55,56,57,58,59,60,61,62,63,64,65,66,67,68,69,70,71,72,73,74,75,76,77,78,79,80,81,82,83,84,85,86,87,88,89,90,91,92,93,94,95,96,97,98,99,100,101,102,103,104,105,106,107,108,109,110,111,112,113,114,115,116,117,118,119,120,121,122,123,124,125,126,127,128,129,130,131,132,133,134,135,136,137]. The detailed study selection flow diagram is shown in Figure 1.

### 3.2. Quality and Characteristics of the Selected Studies

Most of the studies (70%) were classified as studies with a moderate to low risk of bias (quality score ≥5.0 and <8.0 or ≥8.0 and ≤10.0, respectively) while 30% of the studies obtained a low quality score (<5.0) and were considered as a high risk of bias.

The studies were carried out in countries distributed over five continents: Asia (Japan, Korea, China, Taiwan, Thailand, Saudi Arabia, and Iran), North America (USA and Canada) and Latin America (Brazil and Mexico), Europe (Denmark, Finland, The Netherlands, Germany, Poland, UK, Switzerland, Italy, Spain, Portugal, and Greece), Africa (Mauritania, South Africa, and Republic of Mauritius), and Australia. Thus, they were considered representative of a global population. The participants in these studies also represent a mixed population of men and women ranging from young adults to elderly participants, and with a higher prevalence of individuals with a BMI ≥ 25.0 kg/m^2^ (overweight and/or obese volunteers). The quality and depth of reporting of the factors potentially contributing toward inter-individual variability of the effect of flavanols varied among studies. The smoking habits were not reported in most studies but for those studies that did, the participants were typically non-smokers or a mixed sample population. Only two studies [34,57] were carried out specifically with smokers. The total sample population included healthy individuals, overweight and/or obese individuals as well as individuals with an incipient or with a reported chronic risk factor or metabolic disease, comprising principally hypertension, hyperlipidemias, type-2 diabetes, metabolic syndrome, atherosclerosis, coronary artery disease and heart failure. Among these, some participants were taking medication, others were not medicated or medication use was not reported. Studies were selected if the source of flavanols was tea, cocoa, or apple provided as liquid (tea drinks, cocoa beverages, and apple juice) or solid (powder or extracts in capsules, snacks, tablets, and foods) forms. Interventions ranged typically 1–6 months, during which participants followed either a controlled diet or their habitual diets.

### 3.3. Overall Impact of the Supplementation with Flavanol-Containing Tea, Cocoa or Apple Products on Blood Lipids, BMI and WC

The number of RCTs varied in function of the outcome measure studied, from 46 to 120 trials, recruiting a total high number of participants ranging from 2875 to 5931 individuals. Forest plots detailing weighted SDM, SE, 95% confidence intervals and relative weight for the impact of supplementation with flavanol-containing tea, cocoa or apple products on BMI, WC, and blood lipid levels are shown in Figures S1–S6. Visual inspection of the Funnel plots (Figures S7–S12) evidenced symmetrical shapes and absence of publication bias in the case of WC, total cholesterol, LDL, HDL and TAGs. Some asymmetry was however detected for BMI. These results were further confirmed by Egger’s regression. A summary of the random overall effects for each lipid and obesity-related variable, heterogeneity and bias analyses is presented in Table 2.

Despite a high heterogeneity across the studies (*I*^2^ = 70–77% for most variables except for BMI which was more moderate, *I*^2^ = 26.7%), the overall pooled analysis (shown as SDM) significantly confirmed a reduction of BMI (−0.153, *p*-value < 0.001), WC (−0.293, *p*-value < 0.001), blood total cholesterol (−0.214, *p*-value < 0.001), LDL (−0.235, *p*-value < 0.001), and TAGs (−0.114, *p*-value = 0.027). HDL levels were also significantly increased (0.152, *p*-value = 0.005). Sensitivity analyses were carried out using the leave-one-out approach where the meta-analysis was performed with each study removed in turn. The pooled estimates consistently showed a similar effect and significance emphasizing the robustness of these results and that the effect was not driven by any particular study (data not shown). Further support of these results was found by a significant relationship between the duration of the supplementation with the flavanol products and the reduction of WC; total-, LDL- and HDL-cholesterol; and TAGs using random-effects meta-regression analysis. Regression coefficients and *p*-values for each variable can be seen in Table S2.

### 3.4. Analysis of the Potential Factors Influencing Inter-Individual Responses to Flavanols Consumption

#### 3.4.1. Stratification by the Individuals’ Baseline BMI, Sex, Smoking, and Country

Following stratification by the baseline BMI (Table 3), the effects of the flavanol-containing products on BMI, WC, total- and LDL-cholesterol remained significant only in those studies carried out in overweight/obese volunteers (BMI ≥ 25.0 kg/m^2^). HDL-cholesterol levels were also increased in this subgroup (*p* = 0.063) whereas the reducing effects on TAGs levels were not significant in any of the two subgroups. Statistical comparison between ≥25.0 vs. <25.0 kg/m^2^ subgroups did not reach significance for any of the variables investigated. Of note, 61% of the total between-study variance in BMI could be explained by the respective baseline BMI values (total between Q = 2.53, *p*-value = 0.112, R^2^ index = 0.61).

Regarding stratification by sex, the reduction of WC was significant in both men and women after intervention with flavanol-containing products. However, total- and LDL-cholesterol were significantly reduced only in female whereas BMI was significantly lowered only in male. The effects on HDL and TAG levels were no longer significant after stratification by sex (Table 3). Between groups comparison indicated a difference between sexes (total between Q = 2.833, *p*-value = 0.092) and a considerable contribution of the sex to the between-study variance for BMI (R^2^ index = 1.0). 

The reduction of BMI, WC, and total- and LDL-cholesterol in response to the flavanol-containing products was significant in studies carried out in non-smoker volunteers. It was not possible, however, to establish a comparison with habitual smokers due to the very low number of studies carried out with this type of volunteers (*n* = 2 studies). 

Comparison between studies carried out in East Asian countries (assuming Asian ethnicity) against those carried out elsewhere evidenced similar results for BMI, WC, total and LDL cholesterol in both subgroups although the results were slightly less significant in the East Asian subgroup. A small proportion (7%) of the BMI between-groups variance was explained by the study location (East Asian vs. others) (total between Q = 0.963, *p*-value = 0.327, R^2^ index = 0.07). In addition, we found a significant difference in TAG levels in response to flavanol-containing products between the East Asian subgroup and the others with a more pronounced effect in the East Asian studies (total between Q = 7.419, *p*-value = 0.024, R^2^ index < 0.01). When grouping in North America and European countries, we detected a significant reduction of BMI and WC in the Europe group but not in the American one, whereas the LDL-cholesterol reduction resulted significant in the American group only. Statistical comparison between North America and Europe groups showed a difference in the BMI response (total between Q = 3.143, *p*-value = 0.076) and a 28% of the between-group variance explained by this factor (R^2^ index = 0.28). Within Europe, the studies carried out in countries of the Mediterranean area resulted in significant reductions of BMI, WC, total- and LDL-cholesterol and in an increase of HDL (*p* = 0.078). In the non-Mediterranean countries, we only detected a significant reduction of total-cholesterol. Comparison between the two subgroups indicated a significant difference in the WC reduction (Total between Q = 5.228, *p*-value = 0.022, R^2^ index < 0.01).

#### 3.4.2. Stratification by the Individuals’ Medication and Health/Disease Status

The influence of medication on the response to the consumption of the flavanol-containing products was also explored (Table 4). The subgroup including participants without any reported medication showed significant reductions of BMI, WC, total- and LDL-cholesterol as well as a reduction of TAGs (*p* = 0.063). In contrast, in the subgroup of studies including participants under medication the effects did not reach statistical significance. Further comparison between the two subgroups (Yes vs. No medication) revealed no significant differences between them (total between Q = 2.59, *p*-value = 0.107) but 34% of the between-groups variance for the BMI response was explained by this factor (R^2^ index = 0.34).

Regarding health/disease status, participants were stratified as healthy, at risk or with a reported disease. Both in healthy subjects and in participants with a disease, the total- and LDL-cholesterol levels were significantly reduced. Studies conducted with volunteers categorized as at a risk exhibited the most significant reduction of BMI and WC in response to the flavanols. Stratification of the studies by the type of disorder showed a significant reduction of BMI and WC in overweight and/or obese individuals, a significant increase of HDL-cholesterol levels in patients with a dyslipidemia and a significant reduction of LDL-cholesterol in patients with diabetes or hypertension (Table 4). Comparison between each of the subgroups against the healthy subgroup was not significant for any of the variables investigated.

#### 3.4.3. Stratification by the Source/Administration Form of the Flavanols and the Diet during the Intervention

Among the sources of flavanols investigated, our meta-analysis confirmed that supplementation with tea derived products significantly impacts on all the investigated variables except for TAGs (Table 5). Studies carried out with cocoa as the source of flavanols exhibited a significant effect on total-, LDL-cholesterol and TAGs levels whereas intervention with the apple-derived products appears to only modulate total- and LDL-cholesterol levels. Statistical comparison between the sources of flavanols highlighted a significant difference in the effect on BMI between tea and cocoa products (*p*-value = 0.012) with a 29% of the between-groups variance explained by this factor (R^2^ index = 0.29). The apple group resulted also significantly more efficient than the cocoa or tea groups in the reduction of total-cholesterol. In addition, the apple products showed a greater effect on LDL-cholesterol than the tea derived products.

Regarding the supplementation form, the results showed that the administration of tea as solid extracts caused a significant and efficient modulation of all the variables investigated except for HDL and TAGs, whereas the tea beverages were significant at reducing only BMI and LDL-cholesterol (Table 5). Statistical comparison between liquid and solid tea-flavanols administration pointed out at a difference between the two subgroups at reducing LDL cholesterol (*p*-value = 0.096) with 11% of the between-group variance explained by this factor (R^2^ index = 0.11). A very limited number of studies have reported so far the effects of tea purified epigallocatechin gallate (EGCG), one of the main flavanols present in tea. Overall, these studies only support a significant reduction of BMI by this compound. Of note, and as opposed to tea products, the purified EGCG appears to reduce the levels of HDL (results not significant) and increase those of TAGs (*p* = 0.077) (Table 5). Comparison between the EGCG subgroup and the tea drink or the tea extract subgroups indicated that the form of administration (as a purified compound or as a mixture) partially contributed to explaining the between groups variances for HDL (total between Q = 5.211, *p*-value = 0.022, R^2^ index = 0.10, EGCG vs. tea drink; total between Q = 3.835, *p*-value = 0.050, R^2^ index = 0.12, EGCG vs. tea extract) and for TAGs (total between Q = 3.282, *p*-value = 0.070, R^2^ index = 0.06, EGCG vs. tea drink; total between Q = 3.765, *p*-value = 0.052, R^2^ index = 0.09, EGCG vs. tea extract).

Regarding the type of diet (controlled vs. usual) during supplementation with the flavanol-containing products, the reducing effects on BMI, WC, total- and LDL-cholesterol remained significant or marginally significant in both subgroups. The levels of TAGs were also reduced although not significantly. We detected, however, a significant increase in the HDL-cholesterol levels only in the subgroup that followed a controlled diet (Table 5). Further, statistical comparison of the two subgroups highlighted a significant difference on the reduction of WC between them (total between Q = 4.761, *p*-value = 0.029) and a 7% explanation of the between-groups variance by this factor (R^2^ index = 0.07).

#### 3.4.4. Multiple Meta-Regression Analysis of BMI Modulators of the Response to Flavanol-Containing Products Consumption

Multiple meta-regression analysis was performed (Table 6) to derive the independent effect of some of the covariates previously found to partially explain some of the between-groups variance for BMI, i.e., baseline BMI (64%), country where the study was carried out (East-Asian vs. all other countries) (7%), medication use (34%) and source of flavanols (tea vs. cocoa products) (29%). 

Although sex also appeared to contribute greatly to the BMI between-groups variance (R^2^ = 1), it was not included in the multiple regression due to the limited number of studies clearly reporting sex and used in the analysis. The full model reached statistical significance, with a large proportion of variance (94%) accounted for and a considerable number of studies included (*n* = 40 studies). Both medication and source of flavanols were significantly correlated with the reducing effect on BMI, once controlled the influence of the other predictor. In particular, higher effects of the flavanols-products on BMI were found in the absence of the medication and with the consumption of tea products.

## 4. Discussion

The consumption of flavanols may contribute to improve cardiometabolic health via the moderation of a range of associated risk factors. Recent meta-analyses (Table S3) [138,139,140,141,142,143,144,145,146,147,148,149,150,151] suggest that the consumption of flavanol-containing tea and tea products could reduce total- and LDL-cholesterol as well as body mass index (BMI) and waist circumference (WC), while chocolate and cocoa flavanols also appear to regulate blood lipid levels. Nonetheless, the results of these analyses are inconsistent, partly due to the large heterogeneity of the clinical trials included. In addition, some of the anthropometric indicators of obesity such as BMI and WC have not yet been systematically investigated. We herein present the largest meta-analysis investigating the impact of flavanol-containing tea, cocoa and apple products, three major dietary sources of these bioactive compounds [152] on several biomarkers of lipid metabolism and anthropometric variables, such as BMI and WC. Our analysis confirms that the intake of these products is significantly associated with: (1) reduced BMI and WC; and (2) a more favorable lipid profile with a decrease in total- and LDL-cholesterol, and TAG plasma levels, and an increase in HDL-cholesterol levels. In addition, our analyses show that the changes in these biomarkers following consumption of the flavanol-containing products can be influenced by a number of factors and thus, the benefits of these products can significantly vary between specific population subgroups. It is of utmost interest to clarify the impact of these factors in order to discern which population subgroups could most benefit of the intake of these bioactive compounds.

### 4.1. Baseline BMI

There is evidence that baseline BMI may be a potential factor with an impact on the individuals’ response to supplementation with different natural products. For instance, treatment with natural probiotics has been shown to significantly increase HDL only in patients with a baseline BMI ≥ 29 kg/m^2^ [153] or significantly reduce BMI only in participants with a baseline BMI ≥ 25 kg/m^2^ [154]. Regarding flavanol-containing products, a previous meta-analysis of the effects of black tea on blood cholesterol failed to detect differences in the modulation of cholesterol levels between individuals with normal weight or overweight and obese phenotype, but the results of this meta-analysis were estimated using a very small number of trials per subgroup (4 and 5, respectively) [141]. Our stratification approach by baseline BMI provides some evidence that the changes following consumption of flavanol-containing products on BMI, WC and cholesterol levels are more pronounced in individuals with a baseline BMI ≥ 25 kg/m^2^ and supports the fact that supplementation with these products may have a better impact on these risk factors in overweight and/or obese people. Nevertheless, it is not yet clear whether there is a general better efficacy of natural treatments in overweight and/or obese people, or if the effects may vary depending on the biomarkers or the products investigated. More trials in individuals with a normal BMI < 25.0 kg/m^2^ are still needed to further compare and demonstrate significant differences in the benefits of flavanol-containing products in relation to body weight, since most are conducted in populations of greater cardiometabolic risk, who are often obese in nature.

### 4.2. Sex

Understanding the differing responses by sex is becoming increasingly important. Previous work has shown that the reducing effects of green tea on total and LDL-cholesterol were significantly greater in men than in women, giving preliminary evidence of that supplementation with flavanol-containing green tea could have a different effect depending on the sex of the individuals [142]. Our results also support differences between women and men in their capacity to regulate the levels of total and LDL cholesterol in response to the consumption of flavanol-containing products with women exhibiting a more efficient reduction than men. A recent meta-analysis looking at the effects of flavonols (another flavonoids class) on lipids levels, failed to detect a difference between men and women, possibly due to the very low number of trials and participants in the two subgroups [7]. Comparing the regulation of cardiometabolic risk factors between women and men is complex because of the hormonal protection in premenopausal women [155]. We were not able to stratify our analyses based on the age or menopausal status of the women, as these factors were not sufficiently well characterized in the trials selected for the meta-analyses. Nevertheless, our results point out to a different response to flavanols consumption between sexes and reinforce the need to further investigate this factor in future trials specifically designed for this purpose.

### 4.3. Country Where the Study Was Carried Out

Ethno-cultural differences are associated with the risk of development of cardiometabolic disorders [156] and thus, it is important to explore and clarify whether different ethnic groups differ in their responses to consumption of plant bioactives as effective treatment against these diseases. Unfortunately, most of the clinical trials included in the present meta-analysis have not clearly identified the ethnicity of the participants. In the absence of this information, we have explored the potential influence of the country where the studies were carried out. A common comparison is that between studies undertaken in Asian countries vs. non-Asian ones. It has been reported that Asians showed a more marked decrease in the levels of TAGs in response to ϖ-3 fatty acids supplementation as compared to subjects within a USA/European group but, no significant differences were found for total cholesterol or BMI [157]. Flavonols have also been shown to significantly reduce TAGs, total and LDL-cholesterol in studies conducted in Asian countries as compared to those in the EU/European subgroup [7]. Regarding flavanol-containing products, previous meta-analyses have suggested that tea and tea extracts reduce BMI and WC both in Asian and non-Asian trials [140] and that, cocoa products significantly reduce LDL-cholesterol in European countries as compared to USA [149]. Nevertheless, these analyses were all underpowered. Our stratification analysis by country included, in general, a big number of studies per subgroup and showed no apparent differences in the responses to the consumption of tea, cocoa and apple products between East Asian countries and all other countries except for TAGs which were significantly reduced only in the Asian subgroup. We also found some different responses between North American (USA/Canada) and European subgroups, as well as between European Mediterranean and non-Mediterranean ones. This may be partially related to features such as the ethnicity of the participants but also to other factors associated with the life-style of the country. More studies are needed in order to understand the influence of this factor in the response to interventions with plant natural compounds. 

### 4.4. Health and Medication Status

Previous meta-analyses had suggested that the consumption of green tea [146,147], black tea [139], and cocoa products [149] had moderating effects on lipid levels both in healthy subjects and in patients with hyperlipidemia or at a higher cross-over or cross-over or s risk. Other bioactive compounds such as flavonols also had a more pronounced effect in the disease subgroup than in the healthy subgroup as significantly evidenced for LDL-cholesterol [7]. Our results show and corroborate a significant reduction of total- and LDL-cholesterol by the flavanol-containing tea, cocoa and apple products both in healthy participants and in individuals with a disease. On the other hand, BMI and WC were reduced and HDL increased in the three subgroups of healthy, “at risk” and individuals with a disease but the results reached statistical significance in the “at risk” group only. As a whole, these results support a metabolic benefit of the consumption of plant bioactive compounds, and in particular of flavanols, regardless of the health status of the individuals. 

An important consideration regarding the use of plant bioactive compounds as modulators of cardiometabolic risk biomarkers is their potential use as treatment on their own or as coadjuvants in combination with pharmacological drugs [158]. Our results show that the use of flavanol-containing products in the absence of medication was significantly associated with the reduction of BMI, WC, total and LDL cholesterol, as well as TAGs giving some evidence of their efficacy as therapeutics. The number of clinical trials in which the flavanols were supplemented in combination with other drugs was in general smaller than studies carried out in the absence of medication (see Table 4) thus the pooled results did not reach significance. Nonetheless, these data point to a modulatory effect of the flavanol-containing products in medicated individuals. Whether the combined therapy is more efficient and safe than individual treatment with drugs or with natural plant bioactives warrants further investigation.

### 4.5. Source and Form of Administration of the Flavanols

Our results confirm that the flavanol-containing tea products are effective regulators of blood cholesterol (total, LDL and HDL) as well as of BMI and WC. The cocoa or apple products were effective at reducing total- and LDL-cholesterol and the cocoa products were also able to significantly decrease the levels of TAGs. These results might suggest that the metabolic regulatory efficacy of these three flavanol-containing products could be ranked as tea > cocoa > apple but caution should be taken with this interpretation due to the differences in the number of studies carried out with each source of flavanols as well as the differences in the doses and the composition of the products. Further studies are needed to corroborate this comparison. Our analysis also suggests that the administration of tea as a solid extract might be more efficient than tea beverages at reducing WC and total cholesterol. Earlier meta-analyses had suggested that the type of administration of green or black tea either in solid form (extracts and, capsules) or as a drink did not differ at reducing total- and LDL-cholesterol [139,146,147]. Unlike those previous analyses, where the number of studies per subgroup was very small, our stratification between tea drinks and tea extracts included a considerable number of studies per subgroup (>15) and gives preliminary evidence of a potentially higher efficacy of the tea when administered as a solid powder. We may hypothesize that this could be partially related to the presence of higher doses of the bioactive flavanols in such extracts.

### 4.6. Magnitude of the Changes

An interesting issue worth discussing here is the magnitude of the changes attributed to the intake of the flavanol-containing products and to the extent these changes can contribute to the regulation of the analyzed biomarkers in comparison with other approaches, i.e., drugs, lifestyle changes, or other natural compounds. Based on the Cohen guidelines [159], the effects of the flavanol-containing products (expressed as SDM) are, in general, small (≤0.2) or medium (between 0.2 and 0.5) although changes in some specific risk markers in some specific subgroups can be considered high (≥0.8). We used the same random effects model to generate the overall size effects by computing the difference in means (Table S4) and compare these values to some of the reported effects of pharmacological, behavioral or dietary interventions on BMI, WC and lipid levels. Some of the most potent reducing effects on BMI can be achieved with restricted energy diet (−2.7 kg/m2) [160], pharmacological interventions (−1.3 kg/m^2^) [161] or behavioral (diet, exercise) interventions (−0.9 to −1.2 kg/m^2^) [162,163]. These reductions constitute between 5% and 10% of the WHO established limit values for overweight (BMI = 25.0–29.9 kg/m^2^) and obesity (BMI ≥ 30.0 kg/m^2^). Alternatively, intervention with probiotics [154] or nutraceuticals (e.g., lipoic acid) [164] shows a more modest but also significant reduction of BMI (approximately −0.5 kg/m^2^, ~2% change of the WHO values). On average, the size effect of the flavanol-containing products on BMI was smaller (−0.15 kg/m^2^, Table S3) but, notably, this effect may be enhanced in specific subpopulations (up to −0.91 kg/m^2^ in studies conducted in European Mediterranean countries), more similar to other behavioral or dietary interventions. WC can also be significantly and efficiently reduced by brisk walking (−2.83 cm, ~3% of the established 102/88 cm risk values) [162] and, more modestly (−0.53 cm, ~0.5% of the established risk values) by intervention with supplements such as ϖ-3 polyunsaturated fatty acids [165]. Along these lines, intervention with the flavanol-containing products significantly reduces WC by 1.7 cm and can reach reducing values of −4.58 cm in studies conducted in European Mediterranean countries. 

Regarding the cholesterol lowering effects, statins remain, at present, the first-choice agents. The pooled effects of various statins on total- and LDL-cholesterol were −0.89 mmol/L (~17% of the desirable 5.17 mmol/L limit level) and −0.92 mmol/L (~27% of the near optimal 3.36 mmol/L level), respectively [166]. Intervention with natural products such as red yeast rice or spirulina can be as effective as the statins, whereas other plant dietary bioactive compounds such as soluble fiber, sterols/stanols, probiotics and flavonols also significantly reduce total- and LDL-cholesterol by 0.5–0.1 mmol/L [7,167]. In this context, the flavanol-containing products show a similar efficiency at lowering total cholesterol (−0.13 mmol/L) and LDL cholesterol (−0.17 mmol/L). Again, in specific subgroups (e.g., supplementation with flavanol-containing apple products) the reduction of total cholesterol was much more efficient (−0.44 mmol/L). These results are very relevant considering that the reduction of LDL-cholesterol by 1 mmol/L has been associated with a 23% reduction of CVDs risk [168] and reinforce the interest in understanding the influence of different factors on the regulatory efficiency of plant bioactive compounds, in general, and of flavanols in particular.

### 4.7. Additional Recent Evidences

Since the completion of this meta-analysis, additional RCTs investigating the effects of tea or cocoa products containing flavanols on lipid and anthropometric variables have been added to the existing literature [111,169,170,171,172,173,174,175,176,177,178,179,180,181,182,183]. The heterogeneity of these trials remains high with population samples including mixed sexes and ages, obese, overweight, healthy, hyperlipidemic and/or diabetic subjects, etc. The products were administered in different forms, mostly as green tea extracts/capsules or cocoa drinks and at different doses and intervention periods. Some of these studies further support the reduction of total- and LDL-cholesterol by green tea or cocoa flavanols or the increase of HDL by dark chocolate or cocoa [111,169,170,171]. Others show no significant effects on these variables [175]. Noteworthy, some of these trials included stratification analyses by baseline conditions, medication, disease, age, sex, or even genotype and further point to specific responses in some subgroups [169,172,174,176,179]. Likewise, the intake of a cocoa product caused a greater increased of HDL in normocholesterolemic patients than in dyslipidemic patients [176], green tea capsules caused a significant reduction of total-cholesterol in women with a cholesterol baseline value above 5.17 mmol/L [169] or of LDL-cholesterol in patients not receiving anti-hyperlipidemic drugs [179]. Of note, the interactions between two factors: baseline BMI and catechol-*O*-methyltransferase (COMT) genotype, was also recently investigated although the COMT genotype did not modify the effect of green tea extract on any of the variables investigated including BMI [172]. In our study, we were able to identify several factors that may contribute to explaining the heterogeneity on the BMI changes in response to the flavanol-containing products. By multiple meta-regression analysis, we also found that supplementation with these products may be most effective at reducing BMI when specifically using tea products in non-medicated patients. These results highlight the importance of understanding not only the factors affecting the variability in the responses but also the interactions between these factors.

## 5. Conclusions

To the best of our knowledge, the meta-analysis conducted here is the largest one to date that compiles the evidence on the effects on various metabolic risk factors after supplementation with three sources of flavanols, tea, cocoa, and apple products. Our results show consistent and significant modulatory effects on BMI, WC and lipid levels. The size of these effects is modest but similar to that prompted by other natural products. We have also presented evidence of the influence of several factors on these beneficial effects that suggest that flavanols might be very effective in specific subpopulations such as overweight people or non-medicated individuals or when the source of these bioactive compounds is tea. Moreover, a combination of these factors may best explain interindividual variability in the response to the flavanols-containing products.

Although the total number of studies included in the meta-analysis was quite large, the number of studies (and of participants) remained small in some of the subgroup analyses. In addition, many of the studies reported limited or unclear information about the potential factors that may influence the treatment. These limitations affect the capability of the meta-analysis to unequivocally detect moderator variables and limit the significance of our findings. More randomized comparison studies with larger number of well-phenotyped volunteers and providing detailed descriptions of the participants and study characteristics are still needed. This research is crucial for a better understanding of the factors most relevantly involved in the variability of the responses to the consumption of these compounds and to achieve maximum efficacy so that flavanols may become an effective non-pharmacological alternative to battle hyperlipidemia, overweight/obesity and associated cardiometabolic disorders in humans. 

## Figures and Tables

**Table 1:** Potential factors influencing the heterogeneity in the responses to the supplementation with flavanols-containing products investigated in this meta-analysis.

Factors							
Baseline BMI	<25.0 ^a^ (normal and/or underweight)			≥25.0 (overweight and/or obese)			
Sex	Women			Men			
Smoking	Non-smokers			Smokers			
Country where the study was undertaken	East Asian countries (Japan, Korea, China, Thailand, Taiwan)	All other countries	North America (USA, Canada)	European countries			
				Non-Mediterranean countries (Denmark, Finland, The Netherlands, Germany, Poland, UK, Switzerland)		Mediterranean countries (Italy, Spain, Portugal, Greece)	
Medication	Yes			No			
Healthy vs. non-healthy	Healthy individuals ^b^	Individuals at a risk of disease ^c^	Individuals with a reported disease ^d^	Different disorders: overweight and/or obese, dyslipidemia, glucose disorders, blood pressure disorders, mixed ^e^			
Source of flavanols	Cocoa products	Apple products	Tea products	Tea drinks	Tea extracts (capsules, powder)		Tea purified EGCG
Diet during intervention	Controlled diet			Usual diet (includes usual with some restrictions and NR)			

^a^: BMI cut-off values as established by the WHO; ^b^: Includes individuals specifically reported as healthy and not medicated (in some cases medication was not reported, NR); ^c^: Includes individuals not medicated that were overweight and/or obese, or specifically indicated to be borderline, mild condition or at risk of a disease; ^d^: Includes individuals with one or more than one of the following disorders: dyslipidemia, glucose disorders or type-2 diabetes, blood pressure disorders (hypertension), medicated obesity, metabolic syndrome (most cases were also medicated but in some cases medication was NR); ^e^: Individuals reported to have only one of the specified disorders.

**Table 2:** Overall changes (SDM), heterogeneity and publication bias analyses for the impact of flavanol-containing products on BMI, WC and blood lipids levels.

	*n*	N_T_	N_S_	N_C_	SDM	95% CI	Z	*p*-Value	Tau^2^	Q	*df* (*p*-Value)	I^2^ (%)	Egger’s Regression	
													Intercept	*p*-Value (2-Tailed)
BMI	74	4156	2127	2029	−0.153	−0.227, −0.078	−4.009	<0.001	0.027	99.6	73 (0.021)	26.7	1.04	0.024
WC	46	2875	1478	1397	−0.293	−0.438, −0.147	−3.932	<0.001	0.168	156.7	45 (<0.001)	71.3	−1.47	0.101
TC	112	5812	2982	2830	−0.214	−0.328, −0.099	−3.651	<0.001	0.273	479.1	111 (<0.001)	76.8	−0.76	0.226
LDL-C	105	5726	2928	2798	−0.235	−0.345, −0.125	−4.171	<0.001	0.229	408.0	104 (<0.001)	74.5	−0.46	0.459
HDL-C	112	5928	3023	2905	0.152	0.047, 0.256	2.836	0.005	0.214	408.0	111 (<0.001)	72.8	0.72	0.208
TAGs	120	5931	3023	2908	−0.114	−0.215, −0.013	−2.213	0.027	0.209	407.7	119 (<0.001)	70.8	−0.33	0.570

BMI: Body Mass Index; WC: Waist Circumference; TC: Total Cholesterol; LDL-C: Low density Lipoprotein Cholesterol; HDL-C: High Density Lipoprotein Cholesterol; TAGs: Triglycerides; *n*: total number of studies included in the analysis; N_T_: number of total participants; N_S_: number of participants in the supplemented group; N_C_: number of participants in the control group; SDM: standardized difference in means; 95% CI: lower and upper confidence limits for the average SDM: *df*: degrees of freedom; Z: statistic for testing the significance of the average SDM; Tau^2^: between-studies variance; Q: heterogeneity statistic; I^2^: heterogeneity index.

**Table 3:** Stratification analysis of the influence of baseline BMI, sex, smoking and country where the study was carried out on the effects (SDM) on BMI, WC, and blood lipids levels following supplementation with flavanol-containing products.

Factor	Baseline BMI		Sex		Smoking		Country Where the Study Was Undertaken					
Subgroup	<25.0	≥25.0	Women	Men	Non-Smokers	Smokers	East Asian Countries	All Other Countries	North America	All European Countries	European Countries	
											Med Countries	Non-Med Countries
BMI	0.003 ^a^	−0.165	−0.102	−0.321	−0.148	NR	−0.204	−0.126	−0.042	−0.235	−0.306	−0.176
*p*-value	(NS)	(*0.001*)	(NS)	(*0.003*)	(*0.018*)		(*0.001*)	(*0.007*)	(NS)	(*0.001*)	(*0.024*)	(*0.091*)
(*n*, N)	(11, 477)	(42, 2410)	(15, 831)	(8, 339)	(24, 1046)		(23, 1511)	(51, 2645)	(15, 719)	(20, 1051)	(7, 392)	(13, 659)
WC	−0.102	−0.362	−0.643	−0.932	−0.252	NR	−0.217	−0.355	−0.123	−0.573	−1.279	−0.181
*p*-value	(NS)	(*0.000*)	(*0.037*)	(*0.024*)	(*0.008*)		(*0.055*)	(*0.000*)	(NS)	(*0.006*)	(*0.001*)	(NS)
(*n*, N)	(3, 253)	(36, 2002)	(9, 464)	(5, 266)	(16, 750)		(19, 1458)	(27, 1471)	(6, 206)	(13, 757)	(5, 269)	(8, 488)
TC	−0.047	−0.165	−0.493	−0.100	−0.221	0.147	−0.195	−0.223	−0.303	−0.254	−0.255	−0.252
*p*-value	(NS)	(*0.011*)	(*0.012*)	(NS)	(*0.001*)	(NS)	(*0.054*)	(*0.002*)	(*0.027*)	(*0.032*)	(*0.003*)	(*0.003*)
(*n*, N)	(19, 504)	(50, 2863)	(15, 873)	(14, 557)	(41, 1572)	(2, 88)	(37, 2297)	(75, 3515)	(23, 1097)	(30, 1296)	(15, 609)	(15, 639)
LDL-C	−0.062	−0.195	−0.545	−0.131	−0.156	−0.341	−0.236	−0.234	−0.379	−0.185	−0.246	−0.140
*p*-value	(NS)	(*0.002*)	(*0.004*)	(NS)	(*0.039*)	(NS)	(*0.017)*	(*0.001*)	(*0.006*)	(NS)	(*0.042*)	(NS)
(*n*, N)	(13, 396)	(48, 2698)	(15, 868)	(10, 416)	(42, 1764)	(2, 88)	(31, 2334)	(74, 3392)	(25, 1117)	(27, 1176)	(12, 537)	(15, 639)
HDL-C	0.229	0.176	0.226	0.272	0.090	0.152	0.105	0.176	0.286	0.165	0.320	0.029
*p*-value	(NS)	(*0.063*)	(NS)	(NS)	(NS)	(NS)	(NS)	(*0.008*)	(*0.095*)	(NS)	(*0.078*)	(NS)
(*n*, N)	(17, 455)	(52, 2872)	(15, 902)	(16, 634)	(45, 1945)	(2, 88)	(37, 2410)	(75, 3518)	(22, 978)	(34, 1495)	(15, 679)	(18, 816)
TAGs	−0.058	−0.098	−0.067	0.109	−0.051	0.089	−0.196	−0.052	−0.098	−0.185	−0.161	−0.203
p-value	(NS)	(NS)	(NS)	(NS)	(NS)	(NS)	(0.034)	(NS)	(NS)	(0.099)	(NS)	(NS)
(*n*, N)	(23, 596)	(53, 2599)	(16, 887)	(18, 781)	(55, 2243)	(2, 88)	(35, 1975)	(83, 3796)	(23, 921)	(41, 1969)	(18, 939)	(24, 1100)

^a^ Standardized difference in means (SDM); BMI: Body Mass Index; WC: Waist Circumference; TC: Total Cholesterol; LDL-C: Low density Lipoprotein Cholesterol; HDL-C: High Density Lipoprotein Cholesterol; TAGs: Triacylglycerides; Med: Mediterranean; *p*-value < 0.05 was considered significant ; *p*-value < 0.1 and ≥ 0.05 was considered marginally significant ; NS: No significant change/effect; (*n*): Number of studies included; (N): Total number of participants; NR: Not reported.

**Table 4:** Analysis of the influence of medication and health status on the effects (SDM) of the supplementation with flavanols on BMI, WC, and blood lipids levels.

Factor	Medication		Health Status			Type of Disorder			
Subgroup	Yes	No	Healthy Individuals	Individuals at Risk	Individuals with a Disease	Overweight/Obese	Lipid Disorders	Glucose Disorders	Blood Pressure Disorders
BMI	−0.053 ^a^	−0.222	−0.119	−0.195	−0.128	−0.191	−0.063	−0.028	−0.054
*p*-value	(NS)	(0.001)	(NS)	(0.009)	(0.069)	(0.011)	(NS)	(NS)	(NS)
(*n*, N)	(15, 744)	(28, 1312)	(18, 868)	(26, 1753)	(25, 1008)	(22, 1488)	(4, 179)	(5, 265)	(5, 225)
WC	−0.363	−0.445	−0.092	−0.392	−0.169	−0.426	NI	−0.057	NI
*p*-value	(NS)	(0.003)	(NS)	(0.000)	(NS)	(0.000)		(NS)	
(*n*, N)	(5, 309)	(21, 1237)	(4, 250)	(26, 1485)	(9, 466)	(25, 1447)		(4, 188)	
TC	−0.205	−0.296	−0.266	−0.161	−0.241	−0.148	−0.227	−0.372	−0.515
*p*-value	(0.089)	(0.001)	(0.015)	(NS)	(0.014)	(NS)	(NS)	(NS)	(NS)
(*n*, N)	(24, 1174)	(47, 2169)	(38, 1520)	(32, 2243)	(38, 1859)	(24, 1813)	(9, 491)	(8, 463)	(5, 275)
LDL-C	−0.200	−0.289	−0.210	−0.192	−0.311	−0.187	−0.336	−0.579	−0.754
*p*-value	(NS)	(0.004)	(0.028)	(0.061)	(0.003)	(0.082)	(NS)	(0.042)	(0.041)
(*n*, N)	(21, 1020)	(43, 2311)	(37, 1782)	(29, 2049)	(36, 1756)	(23, 1742)	(9, 491)	(6, 408)	(4, 146)
HDL-C	0.162	0.128	0.163	0.229	0.091	−0.062	0.476	0.032	0.115
*p*-value	(NS)	(NS)	(NS)	−0.055	(NS)	(NS)	−0.005	(NS)	(NS)
(*n*, N)	(25, 1212)	(48, 2042)	(37, 1670)	(32, 2187))	(39, 1881)	(20, 1541)	(12, 654)	(8, 488)	(6, 301)
TAGs	−0.165	−0.135	−0.144	−0.08	−0.103	−0.008	−0.258	0.065	−0.251
*p*-value	(NS)	−0.063	(NS)	(NS)	(NS)	(NS)	(NS)	(NS)	(NS)
(*n*, N)	(23, 1132)	(63, 3019)	(45, 2669)	(31, 1205)	(39, 1931)	(24, 912)	(12, 585)	(7, 536)	(6, 250)

^a^ Standardized difference in means (SDM); BMI: Body Mass Index; WC: Waist Circumference; TC: Total Cholesterol; LDL-C: Low density Lipoprotein Cholesterol; HDL-C: High Density Lipoprotein Cholesterol; TAGs: Triacylglycerides; *p*-value < 0.05 was considered significant ; *p*-value < 0.1 and ≥ 0.05 was considered marginally significant ; NS: No significant change/effect; (*n*): Number of studies included; (N): Total number of participants.

**Table 5:** Analysis of the influence of the original source of flavanols and of the diet (during the intervention) on the effects (SDM) of the supplementation on BMI, WC, and blood lipids levels.

Factor	Source of Flavanols						Diet during Supplementation	
Subgroup	Cocoa Products	Apple Products	Tea Products	Tea Drinks	Tea Extracts	Tea Purified EGCG	Controlled	Usual
BMI	0.001 ^a^	−0.11	−0.224	−0.223	−0.212	−0.290	−0.162	−0.149
*p*-value	(NS)	(NS)	(0.000)	−0.007	(0.002)	(0.022)	(0.030)	(0.001)
(*n*, N)	(21, 1014)	(5, 319)	(46, 2704)	(20, 1167)	(26, 1537)	(7, 384)	(23, 1032)	(49, 2990)
WC	−0.106	−0.206	−0.354	−0.22	−0.506	−0.465	−0.500	−0.164
*p*-value	(NS)	(NS)	(0.000)	(NS)	(0.000)	(NS)	(0.000)	(0.084)
(*n*, N)	(8, 430)	(2, 155)	(34, 2228)	(16, 1074)	(17, 1110)	(2, 171)	(18, 903)	(27, 1868)
TC	−0.177	−1.352	−0.143	−0.083	−0.208	−0.051	−0.215	−0.213
*p*-value	−0.018	−0.041	(0.036)	(NS)	(0.008)	(NS)	(0.032)	(0.003)
(*n*, N)	(38, 1625)	(7, 402)	(65, 3666)	(36, 2051)	(26, 1615)	(7, 437)	(39, 1682)	(73, 4130)
LDL-C	−0.252	−0.587	−0.191	−0.119	−0.270	−0.216	−0.184	−0.260
*p*-value	−0.013	−0.007	(0.008)	−0.045	(0.000)	(NS)	(0.062)	(0.000)
(*n*, N)	(35, 1577)	(8, 386)	(61, 3723)	(35, 2235)	(26, 1488)	(7, 206)	(38, 1540)	(67, 4186)
HDL-C	0.16	0.197	0.150	0.17	0.13	−0.291	0.245	0.104
*p*-value	(NS)	(NS)	(0.031)	−0.071	(NS)	(NS)	(0.008)	(NS)
(*n*, N)	(37, 1603)	(7, 386)	(66, 3820)	(38, 2292)	(27, 1484)	(7, 437)	(40, 1759)	(72, 4169)
TAGs	−0.183	−0.173	−0.050	−0.047	−0.053	0.314	−0.139	−0.105
*p*-value	−0.047	(NS)	(NS)	(NS)	(NS)	−0.077	(NS)	(NS)
(*n*, N)	(39, 1691)	(10, 455)	(69, 3700)	(38, 2121)	(30, 1544)	(7, 437)	(46, 1939)	(73, 3922)

^a^ Standardized difference in means (SDM); BMI: Body Mass Index; WC: Waist Circumference; TC: Total Cholesterol; LDL-C: Low density Lipoprotein Cholesterol; HDL-C: High Density Lipoprotein Cholesterol; TAGs: Triacylglycerides; *p*-value < 0.05 was considered significant ; *p*-value < 0.1 and ≥ 0.05 was considered marginally significant ; NS: No significant change/effect; (*n*): Number of studies included; (N): Total number of participants; EGCG: Epigallocatechin gallate.

**Table 6:** Main results of the multiple random-effects meta-regression model for the contribution of the covariates, medication and source of flavanols, on BMI response (Standardized difference in means).

Covariate	Coefficient	SE	95% Lower	95% Upper	Z	*p*-Value
Intercept	−0.0679	0.0899	−0.2440	0.1082	−0.76	0.4500
Medication (No vs. Yes)	0.2481	0.0995	0.0530	0.4431	2.49	0.0127
Source of flavanols (Cocoa vs. Tea)	−0.3278	0.1022	−0.5282	−0.1274	−3.21	0.0013
**Test of the Model**						
Q_R_	15.64					
*df*	2					
*p*-value	0.0004					
R^2^	0.94					
Number of studies included	40 (54% of the total studies used in the meta-analysis)					

SE: standard error for each regression coefficient; *Z*: statistic for testing the statistical significance of each predictor; *P*: probability level; Q_R_: statistic for testing the statistical significance of the full meta-regression model; *df*: degrees of freedom; R^2^: proportion of total between-studies variance explained by the model analog.

## Supplementary Materials

The following are available online at www.mdpi.com/2072-6643/9/7/746/s1. Figure S1: Forest plot of the meta-analysis evaluating the effects of supplementation with flavanols-containing tea, cocoa or apple products on human body mass index (BMI). A total of 74 studies (displayed in alphabetical order) were analysed. Pooled results are shown at the bottom using a random-effects model. SDM: Standardized difference in means, SE: standard error, 95% CI: lower and upper confidence limits for the average SDM, RW: relative weight; Figure S2: Forest plot of the meta-analysis evaluating the effects of supplementation with flavanols-containing tea, cocoa or apple products on human waist circumference (WC). A total of 46 studies (displayed in alphabetical order) were analysed. Pooled results are shown at the bottom using a random-effects model. SDM: standardized difference in means, SE: standard error, 95% CI: lower and upper confidence limits for the average SDM, RW: relative weight; Figure S3: Forest plot of the meta-analysis evaluating the effects of a prolonged supplementation with flavanols-containing tea, cocoa or apple products on human blood levels of total cholesterol. A total of 112 studies (displayed in alphabetical order) were analysed. Pooled results are shown at the bottom using a random-effects model. SDM: standardized difference in means, SE: standard error, 95% CI: lower and upper confidence limits for the average SDM, RW: relative weight; Figure S4: Forest plot of the meta-analysis evaluating the effects of a prolonged supplementation with flavanols-containing tea, cocoa or apple products on human blood levels of LDL cholesterol. A total of 105 studies (displayed in alphabetical order) were analysed. Pooled results are shown at the bottom using a random-effects model. SDM: standardized difference in means, SE: standard error, 95% CI: lower and upper confidence limits for the average SDM, RW: relative weight; Figure S5: Forest plot of the meta-analysis evaluating the effects of a prolonged supplementation with flavanols-containing tea, cocoa or apple products on human blood levels of HDL cholesterol. A total of 112 studies (displayed in alphabetical order) were analysed. Pooled results are shown at the bottom using a random-effects model. SDM: standardized difference in means, SE: standard error, 95% CI: lower and upper confidence limits for the average SDM, RW: relative weight; Figure S6: Forest plot of the meta-analysis evaluating the effects of a prolonged supplementation with flavanols-containing tea, cocoa or apple products on human blood levels of triglycerides (TAGs). A total of 120 studies (displayed in alphabetical order) were analysed. Pooled results are shown at the bottom using a random-effects model. SDM: standardized difference in means, SE: standard error, 95% CI: lower and upper confidence limits for the average SDM, RW: relative weight; Figure S7: Funnel plot and Eager statistics (intercept and 2-tailed *p*-value) of the meta-analysis evaluating the effects of a prolonged supplementation with flavanols-containing tea, cocoa or apple products on human BMI; Figure S8: Funnel plot and Eager statistics (intercept and 2-tailed *p*-value) of the meta-analysis evaluating the effects of a prolonged supplementation with flavanols-containing tea, cocoa or apple products on human WC; Figure S9: Funnel plot and Eager statistics (intercept and 2-tailed *p*-value) of the meta-analysis evaluating the effects of a prolonged supplementation with flavanols-containing tea, cocoa or apple products on human blood levels of total cholesterol; Figure S10: Funnel plot and Eager statistics (intercept and 2-tailed *p*-value) of the meta-analysis evaluating the effects of a prolonged supplementation with flavanols-containing tea, cocoa or apple products on human blood levels of LDL cholesterol; Figure S11: Funnel plot and Eager statistics (intercept and 2-tailed *p*-value) of the meta-analysis evaluating the effects of a prolonged supplementation with flavanols-containing tea, cocoa or apple products on human blood levels of HDL cholesterol; Figure S12: Funnel plot and Eager statistics (intercept and 2-tailed *p*-value) of the meta-analysis evaluating the effects of a prolonged supplementation with flavanols-containing tea, cocoa or apple products on human blood levels of triglycerides (TAGs); Table S1: Mean content (mg/100 fresh weight, FW) in flavonoids of green and black tea infusions, cocoa powder and whole apple illustrative of the composition of the three main sources of flavanols examined in this study: tea, cocoa and apple (data are based on the Phenol-Explorer database); Table S2: Results of the meta-regression of the changes in BMI, WC and blood lipids levels vs. duration of the supplementation with the flavanol-containing tea, cocoa or apple products; Table S3: Summary of most recent meta-analysis looking at the effects of flavanol-containing tea or cocoa products on anthropometric measurements and blood lipids associated with the development of metabolic disorders; Table S4: Overall effect size estimations (DM) for the impact of flavanols containing products on BMI, WC and blood lipids levels.
