# Supplementary material for: A Systematic Review and Meta-Analysis of the Effects of Flavanol-Containing Tea, Cocoa and Apple Products on Body Composition and Blood Lipids: Exploring the Factors Responsible for Variability in Their Efficacy
Source: Nutrients. 2017 Jul 13;9(7):746. doi: 10.3390/nu9070746 (PMC5537860; doi:10.3390/nu9070746)
Supplement: Supplementary file 1 [file nutrients-09-00746-s001.zip › Figure S1.pdf]

Figure S1.- Forest plot of the meta-analysis evaluating the effects of supplementation with flavanols-containing tea, cocoa or apple products on human body mass index (BMI). A total of 74 studies (displayed in alphabetical order) were analysed. Pooled results are shown at the bottom using a random-effects model. SDM: Standardized difference in means, SE: standard error, 95% CI: lower and upper confidence limits for the average SDM, RW: relative weight.

First author & year	SDM	SE	95% CI		RW
Akazome 2010	0.000	0.365	-0.716	0.716	0.93
Akazome 2010	-0.129	0.212	-0.545	0.287	1.98
Al-Faris 2008	0.000	0.260	-0.510	0.510	1.54
Auvichayapat 2008	-0.622	0.266	-1.142	-0.101	1.49
Barth 2012	-0.029	0.244	-0.508	0.450	1.67
Batista 2009	-0.721	0.359	-1.425	-0.016	0.95
Belcaro 2013	-1.072	0.216	-1.495	-0.648	1.94
Bogdanski 2012	-0.035	0.267	-0.559	0.489	1.48
Bøhn 2014	-0.054	0.228	-0.501	0.393	1.82
Bondia-Pons 2014	0.000	0.316	-0.620	0.620	1.16
Brown 2009	-0.841	0.223	-1.278	-0.405	1.88
Cardoso 2013	-1.412	0.527	-2.444	-0.379	0.50
Chai 2012	-0.206	0.202	-0.601	0.189	2.11
Chan 2012	0.037	0.156	-0.269	0.343	2.71
Chen 2016	-0.133	0.228	-0.580	0.314	1.82
Crews 2008	-0.034	0.211	-0.447	0.379	2.00
Davison 2008	0.054	0.392	-0.715	0.823	0.83
Davison 2008	0.239	0.419	-0.582	1.060	0.74
de Jesus Romero-Prado 2015	-0.090	0.225	-0.532	0.351	1.85
Diepvens 2006	-0.035	0.295	-0.613	0.543	1.29
Frank 2009	-0.062	0.348	-0.744	0.621	1.00
Fu 2011	-0.382	0.451	-1.266	0.503	0.65
Fujita 2008	-0.030	0.292	-0.603	0.543	1.31
Fukino 2008	-0.386	0.261	-0.897	0.124	1.53
Grassi 2005a	0.000	0.447	-0.877	0.877	0.66
Grassi 2005b	0.000	0.518	-1.014	1.014	0.51
Hodgson 2002	0.000	0.437	-0.856	0.856	0.69
Hsu 2006	-0.035	0.577	-1.167	1.097	0.42
Hsu 2008	0.024	0.227	-0.420	0.469	1.83
Hsu 2011	-0.319	0.244	-0.798	0.160	1.67
Ichinose 2011	-0.189	0.579	-1.323	0.945	0.42
Kajimoto 2005	-0.169	0.175	-0.512	0.174	2.44
Kajimoto 2005	-0.097	0.175	-0.439	0.246	2.44
Kajimoto 2003	0.075	0.258	-0.431	0.582	1.55
Kovacs 2004	-0.178	0.197	-0.563	0.207	2.16
Liu 2014	-0.131	0.228	-0.578	0.316	1.82
Mähler 2015	-0.032	0.632	-1.272	1.207	0.36
Mähler 2015	-0.229	0.709	-1.620	1.161	0.29
Mastroiacovo 2015	-0.056	0.265	-0.575	0.464	1.50
Mastroiacovo 2015	-0.039	0.265	-0.559	0.480	1.50
Matsuyama 2008	0.459	0.329	-0.185	1.103	1.10
Mielgo-Ayuso 2014	-0.021	0.220	-0.452	0.409	1.91
Mirzaei 2009	-0.063	0.223	-0.499	0.374	1.88
Miyazaki 2013	0.000	0.283	-0.554	0.554	1.37
Mogollon 2013	0.035	0.309	-0.570	0.641	1.20
Monagas 2009	-0.648	0.317	-1.269	-0.028	1.16
Mousavi 2013	-0.061	0.334	-0.716	0.593	1.07
Mousavi 2013	-0.071	0.336	-0.731	0.588	1.06
Muniyappa 2008	-0.143	0.317	-0.763	0.478	1.16
Murphy 2003	0.000	0.379	-0.743	0.743	0.88
Nagao 2005	-0.159	0.339	-0.823	0.505	1.05
Nagao 2007	-1.000	0.137	-1.268	-0.732	3.00
Nagao 2009	-0.025	0.306	-0.624	0.574	1.22
Nickols-Richardson 2014	-0.429	0.285	-0.988	0.130	1.35
Njike 2011	0.780	0.332	0.129	1.432	1.08
Rull 2015	-0.109	0.393	-0.878	0.661	0.83
Sansone 2015	-0.204	0.201	-0.597	0.189	2.12
Sone 2011	-0.050	0.280	-0.599	0.499	1.39
Stendell-Hollis 2010	-0.153	0.326	-0.792	0.486	1.11
Suliburska 2012	-0.109	0.267	-0.633	0.415	1.48
Takahashi 2014	0.000	0.426	-0.836	0.836	0.72
Taubert 2007	0.013	0.302	-0.579	0.604	1.25
Toolsee 2013	0.018	0.259	-0.489	0.525	1.55
Toolsee 2013	0.048	0.252	-0.446	0.543	1.60
Tzounis 2011	-0.048	0.447	-0.925	0.829	0.66
Velliquette 2015	-0.032	0.354	-0.726	0.662	0.98
Vieira Senger 2012	-0.067	0.299	-0.652	0.519	1.26
Vlachopoulos 2005	0.000	0.437	-0.856	0.856	0.69
West 2014	0.073	0.365	-0.643	0.789	0.93
Widmer 2013	-0.131	0.278	-0.677	0.415	1.40
Wu 2012	-0.251	0.237	-0.715	0.213	1.74
Wu 2012	-0.214	0.238	-0.681	0.252	1.73
Yang 2012	-0.224	0.366	-0.942	0.494	0.92
Yang 2014	0.086	0.239	-0.383	0.555	1.72
**Intervention effect**	**-0.153**	**0.039**	**-0.227**	**-0.078**	

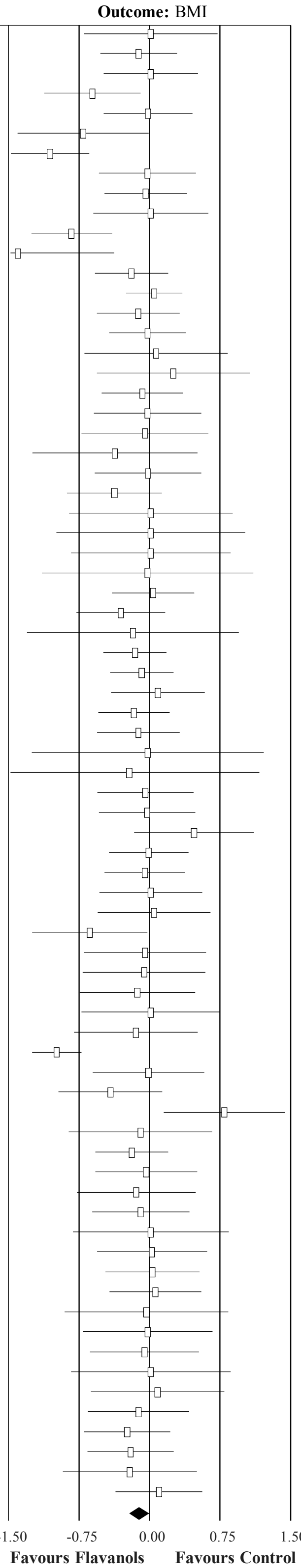

Test for overall effect Z=-4.009; *P*=0.000; F=2127, C=2029
Heterogeneity Tau^2=0.027; Q=99.6; df=73(*P*=0.021); I^2=26.7
