# Supplementary material for: A Systematic Review and Meta-Analysis of the Effects of Flavanol-Containing Tea, Cocoa and Apple Products on Body Composition and Blood Lipids: Exploring the Factors Responsible for Variability in Their Efficacy
Source: Nutrients. 2017 Jul 13;9(7):746. doi: 10.3390/nu9070746 (PMC5537860; doi:10.3390/nu9070746)
Supplement: Supplementary file 1 [file nutrients-09-00746-s001.zip › Figure S2.pdf]

Figure S2.- Forest plot of the meta-analysis evaluating the effects of supplementation with flavanols-containing tea, cocoa or apple products on human waist circumference (WC). A total of 46 studies (displayed in alphabetical order) were analysed. Pooled results are shown at the bottom using a random-effects model. SDM: standardized difference in means, SE: standard error, 95% CI: lower and upper confidence limits for the average SDM, RW: relative weight.

First author & year	SDM	SE	95% CI		RW
Akazome 2010	-0.379	0.216	-0.803	0.045	2.57
Auvichayapat 2008	-0.420	0.265	-0.940	0.099	2.32
Bajerska 2015	-0.054	0.302	-0.645	0.538	2.13
Barth 2012	0.000	0.244	-0.479	0.479	2.43
Basu 2011	0.123	0.429	-0.717	0.963	1.57
Basu 2011	0.031	0.400	-0.753	0.816	1.69
Belcaro 2013	-2.831	0.400	-3.615	-2.047	1.69
Belcaro 2013	-4.014	0.496	-4.987	-3.042	1.34
Bogdanski 2012	-0.032	0.267	-0.556	0.491	2.31
Bøhn 2014	-0.108	0.228	-0.555	0.340	2.51
Bondia-Pons 2014	0.000	0.316	-0.620	0.620	2.06
Brown 2009	-0.778	0.221	-1.212	-0.345	2.55
Cardoso 2013	-2.049	0.582	-3.190	-0.908	1.09
Chan 2012	-0.027	0.153	-0.327	0.272	2.89
Chen 2016	-0.210	0.229	-0.658	0.238	2.51
Davison 2008	0.012	0.392	-0.756	0.781	1.72
Davison 2008	0.044	0.417	-0.774	0.862	1.62
Diepvens 2006	-0.058	0.295	-0.636	0.520	2.17
Hsu 2008	0.040	0.227	-0.405	0.484	2.52
Hsu 2011	-0.235	0.243	-0.712	0.242	2.43
Ibero-Baraibar 2014	-0.104	0.292	-0.676	0.468	2.18
Kajimoto 2005	-0.091	0.175	-0.434	0.251	2.79
Kajimoto 2005	-0.037	0.175	-0.380	0.305	2.79
Kovacs 2004	-0.278	0.197	-0.664	0.108	2.67
Liu 2014	0.225	0.229	-0.224	0.673	2.51
Matsuyama 2008	-0.086	0.325	-0.723	0.550	2.02
Mielgo-Ayuso 2014	-0.153	0.220	-0.584	0.278	2.56
Miyazaki 2013	-0.137	0.283	-0.692	0.418	2.23
Mousavi 2013	-0.163	0.334	-0.818	0.492	1.98
Mousavi 2013	-0.305	0.338	-0.968	0.358	1.96
Nagao 2005	-0.212	0.340	-0.877	0.454	1.95
Nagao 2007	-0.279	0.130	-0.533	-0.024	2.99
Nagao 2009	-0.124	0.306	-0.724	0.477	2.11
Nickols-Richardson 2014	-0.627	0.289	-1.195	-0.060	2.20
Njike 2011	-0.129	0.321	-0.758	0.499	2.04
Oi 2015	-0.225	0.334	-0.880	0.430	1.98
Rull 2015	-0.107	0.393	-0.876	0.662	1.72
Sone 2011	-0.042	0.280	-0.591	0.507	2.24
Stendell-Hollis 2010	-0.228	0.327	-0.868	0.412	2.01
Suliburska 2012	-0.107	0.267	-0.631	0.417	2.31
Takahashi 2014	-0.196	0.427	-1.034	0.641	1.58
Tsai 2009	-1.127	0.202	-1.522	-0.731	2.65
Vieira Senger 2012	-0.178	0.299	-0.764	0.409	2.15
Wang 2010	-0.083	0.211	-0.497	0.330	2.60
West 2014	0.169	0.366	-0.548	0.886	1.83
Yang 2012	-0.502	0.371	-1.229	0.225	1.81
**Intervention effect**	**-0.293**	**0.074**	**-0.438**	**-0.147**	

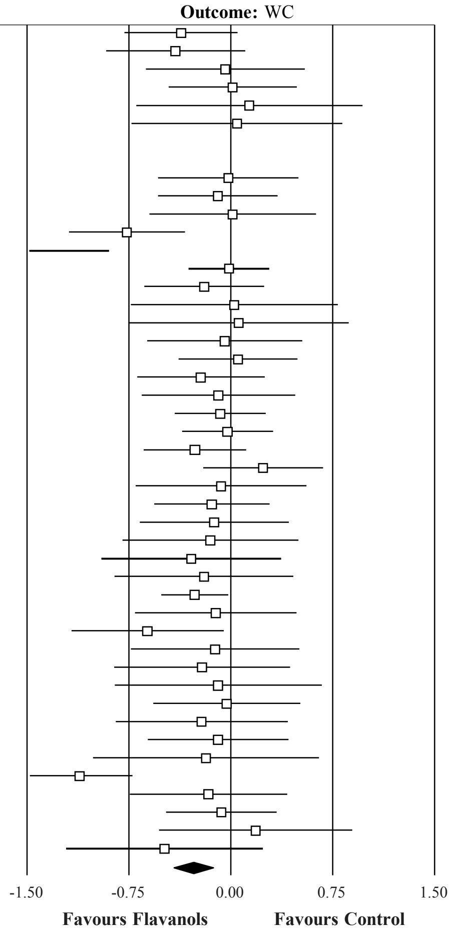

Test for overall effect Z=-3.932; P=0.000; F=1478, C=1397
Heterogeneity Tau^2=0.168; Q=156.7; df=45(P=0.000); I^2=71.3
