# Supplementary material for: A Systematic Review and Meta-Analysis of the Effects of Flavanol-Containing Tea, Cocoa and Apple Products on Body Composition and Blood Lipids: Exploring the Factors Responsible for Variability in Their Efficacy
Source: Nutrients. 2017 Jul 13;9(7):746. doi: 10.3390/nu9070746 (PMC5537860; doi:10.3390/nu9070746)
Supplement: Supplementary file 1 [file nutrients-09-00746-s001.zip › Figure S3.pdf]

Figure S3.- Forest plot of the meta-analysis evaluating the effects of a prolonged supplementation with flavanols-containing tea, cocoa or apple products on human blood levels of total cholesterol. A total of 112 studies (displayed in alphabetical order) were analysed. Pooled results are shown at the bottom using a random-effects model. SDM: standardized difference in means, SE: standard error, 95% CI: lower and upper confidence limits for the average SDM, RW: relative weight.

First author & year	SDM	SE	95% CI		RW
Akazome 2010	0.184	0.366	-0.534	0.901	0.84
Akazome 2010	-0.132	0.215	-0.553	0.289	1.07
Al-Faris 2008	0.197	0.261	-0.315	0.708	1.00
Al-Faris 2008	0.000	0.260	-0.510	0.510	1.00
Baba 2007a	-0.142	0.401	-0.927	0.644	0.79
Baba 2007b	-0.099	0.224	-0.537	0.340	1.06
Baba 2007b	-0.014	0.224	-0.452	0.424	1.06
Baba 2007b	-0.014	0.224	-0.452	0.425	1.06
Bahorun 2012	0.457	0.354	-0.237	1.151	0.86
Bahorun 2012	0.003	0.376	-0.734	0.740	0.83
Balzer 2008	-0.471	0.317	-1.092	0.150	0.92
Barth 2012	0.047	0.244	-0.432	0.526	1.03
Basu 2011	-0.065	0.428	-0.905	0.774	0.75
Basu 2011	-0.512	0.407	-1.309	0.286	0.78
Bingham 1997	-0.047	0.283	-0.601	0.508	0.97
Bogdanski 2012	-0.556	0.272	-1.089	-0.022	0.99
Bøhn 2014	0.099	0.228	-0.348	0.546	1.05
Bondia-Pons 2014	-0.187	0.317	-0.809	0.434	0.92
Brown 2009	0.038	0.213	-0.380	0.456	1.07
Brown 2011	-0.208	0.247	-0.692	0.276	1.03
Chai 2012	-4.819	0.398	-5.599	-4.039	0.79
Chen 2016	-0.494	0.231	-0.948	-0.041	1.05
Coimbra 2006	-0.174	0.372	-0.904	0.556	0.83
Crew 2015	-0.395	0.407	-1.193	0.403	0.78
Crews 2008	-0.111	0.211	-0.524	0.303	1.08
Davies 2003	-0.899	0.543	-1.963	0.165	0.60
Davison 2008	0.673	0.403	-0.118	1.463	0.79
Davison 2008	0.375	0.421	-0.451	1.200	0.76
de Jesus Romero-Prado 2015	-0.631	0.231	-1.083	-0.179	1.05
De la Torre 2014	-0.137	0.374	-0.870	0.595	0.83
Desideri 2012	-0.434	0.263	-0.951	0.082	1.00
Desideri 2012	-0.409	0.265	-0.929	0.111	1.00
Diepvens 2006	-1.857	0.390	-2.621	-1.093	0.81
Dower 2015	-0.125	0.339	-0.789	0.539	0.88
Duffy 2001	-0.123	0.401	-0.909	0.662	0.79
Erba 2005	-0.098	0.447	-0.975	0.779	0.72
Farouque 2006	0.402	0.328	-0.240	1.044	0.90
Flammer 2012	0.000	0.316	-0.620	0.620	0.92
Frank 2009	-0.177	0.349	-0.861	0.507	0.87
Freese 1999	-0.403	0.452	-1.289	0.482	0.72
Fu 2011	-0.994	0.474	-1.923	-0.065	0.69
Fujita 2008	-0.518	0.298	-1.102	0.066	0.95
Fukino 2008	-0.076	0.365	-0.792	0.641	0.84
Grassi 2005a	-0.614	0.458	-1.510	0.283	0.71
Grassi 2005b	0.250	0.520	-0.768	1.268	0.63
Hodgson 2002	-0.308	0.440	-1.170	0.553	0.73
Hodgson 2013	-0.131	0.230	-0.581	0.319	1.05
Hsu 2006	0.101	0.578	-1.032	1.233	0.56
Hsu 2008	-0.172	0.227	-0.617	0.273	1.05
Hsu 2011	1.069	0.259	0.561	1.578	1.01
Ibero-Baraibar 2014	-0.194	0.292	-0.768	0.379	0.95
Inami 2007	-0.294	0.356	-0.991	0.403	0.86
Ishikawa 1997	-0.333	0.446	-1.207	0.541	0.73
Kajimoto 2005	0.105	0.176	-0.239	0.449	1.13
Kajimoto 2005	-0.060	0.177	-0.408	0.287	1.12
Kajimoto 2005	0.045	0.175	-0.299	0.389	1.13
Kajimoto 2005	-0.181	0.176	-0.527	0.165	1.13
Kajimoto 2003	-0.461	0.262	-0.974	0.053	1.00
Koutelidakis 2014	-0.065	0.306	-0.665	0.534	0.93
Lettieri-Barbato 2012	0.146	0.518	-0.870	1.162	0.63
Liu 2014	-0.086	0.228	-0.533	0.361	1.05
Maki 2009	-0.332	0.195	-0.714	0.050	1.10
Mastroiacovo 2015	-0.502	0.269	-1.029	0.026	0.99
Mastroiacovo 2015	-0.416	0.268	-0.941	0.109	0.99
Matsuyama 2008	-0.153	0.325	-0.790	0.484	0.90
Mellor 2010	-0.072	0.578	-1.204	1.060	0.56
Mielgo-Ayuso 2014	-0.260	0.221	-0.692	0.172	1.06
Miyazaki 2013	-0.350	0.285	-0.909	0.208	0.97
Monagas 2009	-0.257	0.310	-0.864	0.350	0.93
Mousavi 2013	-0.270	0.335	-0.927	0.387	0.89
Mousavi 2013	-0.382	0.339	-1.047	0.283	0.88
Mozaffari-Khosravi 2009	0.567	0.280	0.017	1.116	0.97
Muniyappa 2008	-0.111	0.742	-1.565	1.343	0.42
Nagao 2005	0.172	0.339	-0.492	0.836	0.88
Nagao 2007	-0.136	0.129	-0.389	0.118	1.18
Nagao 2009	0.000	0.306	-0.599	0.599	0.93
Nagasako-Akazome 2007	-5.148	0.670	-6.462	-3.834	0.47
Nantz 2009	-0.240	0.191	-0.613	0.133	1.11
Nickols-Richardson 2014	-0.392	0.283	-0.947	0.163	0.97
Njike 2011	1.135	0.345	0.458	1.811	0.87
Oi 2015	0.399	0.337	-0.262	1.059	0.89
Osakabe 2004	-1.106	0.759	-2.595	0.382	0.40
Osakabe 2004	0.459	0.716	-0.945	1.863	0.44
Osakabe 2004	-0.066	0.707	-1.453	1.320	0.44
Parsaeyan 2014	-2.916	0.287	-3.479	-2.353	0.96
Pierin Batista 2009	0.526	0.354	-0.169	1.220	0.86
Rostami 2015	-0.108	0.259	-0.616	0.399	1.01
Ryu 2006	-0.030	0.270	-0.559	0.499	0.99
Sansone 2015	-0.116	0.200	-0.508	0.277	1.09
Shiina 2009	-0.065	0.320	-0.693	0.564	0.91
Shimada 2004	-0.566	0.435	-1.418	0.287	0.74
Sone 2011	0.163	0.281	-0.387	0.713	0.97
Stendell-Hollis 2010	-0.216	0.326	-0.856	0.424	0.90
Taubert 2007	-0.688	0.341	-1.357	-0.020	0.88
Toolsee 2013	-0.168	0.259	-0.676	0.341	1.01
Toolsee 2013	-0.222	0.253	-0.718	0.274	1.02
Troup 2015	0.808	0.276	0.268	1.348	0.98
Tsai 2009	-1.592	0.215	-2.013	-1.170	1.07
Tzounis 2011	-0.253	0.449	-1.133	0.627	0.72
Vafa 2011	-0.017	0.295	-0.595	0.561	0.95
Velliquette 2015	-0.055	0.354	-0.749	0.639	0.86
Vieira Senger 2012	-0.150	0.299	-0.736	0.437	0.94
Villano 2010	-0.028	0.542	-1.090	1.033	0.60
Vlachopoulos 2005	0.154	0.438	-0.704	1.012	0.74
West 2014	-0.032	0.365	-0.747	0.684	0.84
Westphal 2011	0.333	0.476	-0.600	1.267	0.68
Widlansky 2007	-0.031	0.309	-0.636	0.574	0.93
Widmer 2013	-0.262	0.279	-0.810	0.285	0.98
Wu 2012	0.431	0.239	-0.037	0.898	1.04
Wu 2012	-0.184	0.238	-0.651	0.282	1.04
Yang 2012	-0.156	0.366	-0.874	0.561	0.84
Yang 2014	-0.006	0.239	-0.474	0.463	1.04
**Intervention effect**	**-0.214**	**0.059**	**-0.328**	**-0.099**	

Outcome: Total cholesterol

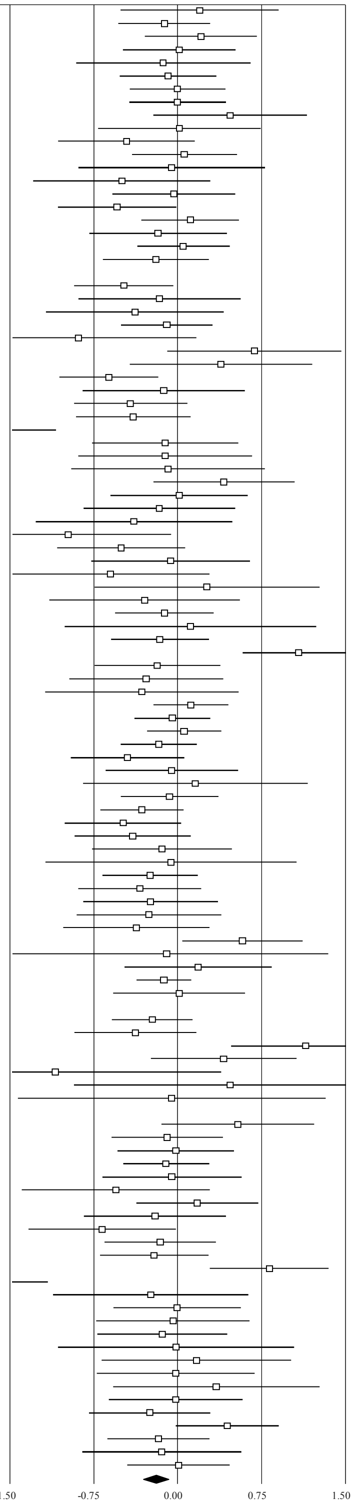

Favours Flavanols **Favours Control**

Test for overall effect Z=-3.651; P=0.000; F=2982, C=2830

Heterogeneity Tau^2=0.273; Q=479.1; df=111(P=0.000); I^2=76.8
