# Supplementary material for: A Systematic Review and Meta-Analysis of the Effects of Flavanol-Containing Tea, Cocoa and Apple Products on Body Composition and Blood Lipids: Exploring the Factors Responsible for Variability in Their Efficacy
Source: Nutrients. 2017 Jul 13;9(7):746. doi: 10.3390/nu9070746 (PMC5537860; doi:10.3390/nu9070746)
Supplement: Supplementary file 1 [file nutrients-09-00746-s001.zip › Figure S4.pdf]

Figure S4.- Forest plot of the meta-analysis evaluating the effects of a prolonged supplementation with flavanols-containing tea, cocoa or apple products on human blood levels of LDL cholesterol. A total of 105 studies (displayed in alphabetical order) were analysed. Pooled results are shown at the bottom using a random-effects model. SDM: standardized difference in means, SE: standard error, 95% CI: lower and upper confidence limits for the average SDM, RW: relative weight.

First author & year	SDM	SE	95% CI		RW
Akazome 2010	0.028	0.365	-0.687	0.744	0.87
Akazome 2010	-0.134	0.215	-0.555	0.286	1.15
Al-Faris 2008	0.391	0.263	-0.124	0.906	1.06
Al-Faris 2008	0.000	0.260	-0.510	0.510	1.07
Baba 2007a	-0.352	0.403	-1.143	0.438	0.81
Baba 2007b	-0.197	0.224	-0.636	0.242	1.13
Baba 2007b	-0.093	0.224	-0.531	0.346	1.14
Baba 2007b	-0.137	0.224	-0.576	0.301	1.13
Bahorun 2012	-0.474	0.354	-1.168	0.221	0.89
Bahorun 2012	0.086	0.376	-0.651	0.824	0.86
Balzer 2008	-0.187	0.313	-0.800	0.427	0.97
Barth 2012	-0.220	0.245	-0.700	0.260	1.10
Basu 2011	-0.221	0.429	-1.063	0.621	0.77
Basu 2011	-0.029	0.400	-0.814	0.755	0.81
Batista 2009	-0.201	0.349	-0.885	0.483	0.90
Bingham 1997	-0.025	0.283	-0.580	0.529	1.03
Bogdanski 2012	-0.420	0.270	-0.950	0.109	1.05
Bøhn 2014	0.107	0.228	-0.340	0.555	1.13
Bondia-Pons 2014	-0.000	0.316	-0.620	0.620	0.96
Brown 2011	-0.386	0.248	-0.873	0.101	1.09
Chai 2012	-4.110	0.358	-4.812	-3.409	0.89
Chen 2016	-0.498	0.231	-0.951	-0.044	1.12
Coimbra 2006	-0.361	0.375	-1.095	0.373	0.86
Crews 2008	-0.178	0.211	-0.592	0.236	1.16
Davies 2003	-0.825	0.539	-1.881	0.232	0.61
Davison 2008	0.065	0.392	-0.704	0.834	0.83
Davison 2008	2.332	0.541	1.272	3.392	0.61
De la Torre 2014	-0.123	0.374	-0.856	0.609	0.86
Desideri 2012	-0.551	0.265	-1.071	-0.031	1.06
Desideri 2012	-0.469	0.266	-0.991	0.054	1.06
Diepvens 2006	-0.157	0.295	-0.736	0.422	1.00
Dower 2015	0.000	0.338	-0.663	0.663	0.92
Duffy 2001	0.000	0.400	-0.785	0.785	0.81
Erba 2005	-0.188	0.448	-1.066	0.691	0.74
Farouque 2006	-0.703	0.334	-1.358	-0.048	0.93
Flammer 2012	1.018	0.336	0.359	1.677	0.93
Fu 2011	-1.587	0.513	-2.592	-0.582	0.64
Fujita 2008	-0.400	0.296	-0.981	0.180	1.00
Fukino 2008	-0.084	0.365	-0.800	0.632	0.87
Grassi 2005a	-0.724	0.462	-1.629	0.181	0.72
Grassi 2005b	0.000	0.518	-1.014	1.014	0.64
Hodgson 2002	-0.154	0.438	-1.012	0.704	0.75
Hsu 2008	-0.326	0.228	-0.774	0.121	1.13
Hsu 2011	-0.189	0.243	-0.666	0.288	1.10
Ibero-Baraibar 2014	-0.003	0.292	-0.575	0.569	1.01
Inami 2007	-0.286	0.356	-0.983	0.411	0.89
Kajimoto 2005	0.000	0.175	-0.344	0.344	1.22
Kajimoto 2005	-0.329	0.177	-0.677	0.019	1.22
Kajimoto 2005	0.000	0.175	-0.344	0.344	1.22
Kajimoto 2005	-0.224	0.177	-0.570	0.122	1.22
Kajimoto 2003	-1.689	0.354	-2.382	-0.995	0.89
Koutelidakis 2014	0.218	0.307	-0.383	0.819	0.98
Liu 2014	-0.213	0.229	-0.661	0.235	1.13
Maki 2009	-0.104	0.194	-0.484	0.275	1.19
Mastroiacovo 2015	-0.547	0.270	-1.076	-0.018	1.05
Mastroiacovo 2015	-0.447	0.268	-0.972	0.079	1.05
Matsuyama 2008	-0.213	0.325	-0.851	0.425	0.95
Mielgo-Ayuso 2014	-0.197	0.220	-0.629	0.234	1.14
Miyazaki 2013	-0.269	0.284	-0.826	0.288	1.02
Monahan 2012	0.218	0.419	-0.603	1.038	0.78
Monahan 2012	-0.303	0.420	-1.126	0.520	0.78
Monahan 2012	-0.155	0.418	-0.974	0.664	0.78
Monahan 2012	0.131	0.418	-0.688	0.950	0.79
Mousavi 2013	0.264	0.335	-0.393	0.921	0.93
Mousavi 2013	-0.173	0.337	-0.833	0.487	0.92
Mozaffari-Khosravi 2009	0.329	0.277	-0.214	0.871	1.04
Muniyappa 2008	-0.201	0.742	-1.656	1.254	0.41
Nagao 2005	-0.351	0.341	-1.019	0.317	0.92
Nagao 2007	-0.171	0.129	-0.424	0.083	1.29
Nantz 2009	-0.061	0.190	-0.433	0.311	1.19
Nickols-Richardson 2014	-1.242	0.318	-1.865	-0.618	0.96
Oi 2015	0.326	0.336	-0.332	0.984	0.93
Parsaeyan 2014	-3.121	0.298	-3.705	-2.537	1.00
Ravn-Haren 2013	-0.545	0.546	-1.615	0.525	0.60
Ravn-Haren 2013	1.553	0.867	-0.146	3.253	0.32
Rostami 2015	-0.082	0.259	-0.590	0.425	1.07
Rull 2015	-0.316	0.395	-1.090	0.457	0.82
Ryu 2006	-0.122	0.270	-0.651	0.407	1.05
Sansone 2015	-0.202	0.201	-0.595	0.191	1.18
Shiina 2009	-0.061	0.320	-0.689	0.567	0.96
Shimada 2004	-0.307	0.429	-1.147	0.534	0.77
Stendell-Hollis 2010	-0.327	0.328	-0.969	0.315	0.94
Suliburska 2012	-0.316	0.269	-0.843	0.211	1.05
Taubert 2007	-6.385	1.608	-9.536	-3.233	0.11
Toolsee 2013	-0.239	0.260	-0.748	0.270	1.07
Toolsee 2013	-0.209	0.253	-0.705	0.286	1.08
Troup 2015	1.341	0.293	0.766	1.916	1.01
Tsai 2009	-1.146	0.202	-1.542	-0.749	1.17
Tzounis 2011	-0.266	0.449	-1.146	0.614	0.74
Vafa 2011	-0.088	0.295	-0.667	0.490	1.00
Velliquette 2015	-0.084	0.354	-0.779	0.611	0.89
Vieira Senger 2012	-0.161	0.299	-0.748	0.425	0.99
Vlachopoulos 2005	-0.603	0.447	-1.479	0.272	0.74
Wang 2000	0.161	0.211	-0.253	0.576	1.16
Wang 2000	0.000	0.209	-0.410	0.410	1.16
Wang 2010	-0.117	0.216	-0.541	0.306	1.15
Wang 2010	-0.163	0.209	-0.573	0.247	1.16
West 2014	-0.073	0.365	-0.789	0.643	0.87
Westphal 2011	0.500	0.479	-0.438	1.438	0.69
Widlansky 2007	-0.104	0.309	-0.710	0.501	0.98
Widmer 2013	-0.163	0.279	-0.709	0.383	1.03
Wu 2012	-0.557	0.240	-1.028	-0.086	1.10
Wu 2012	-0.082	0.236	-0.544	0.381	1.11
Yang 2012	-0.019	0.365	-0.735	0.697	0.87
Yang 2014	-0.239	0.240	-0.709	0.232	1.11
**Intervention effect**	**-0.235**	**0.056**	**-0.345**	**-0.125**	

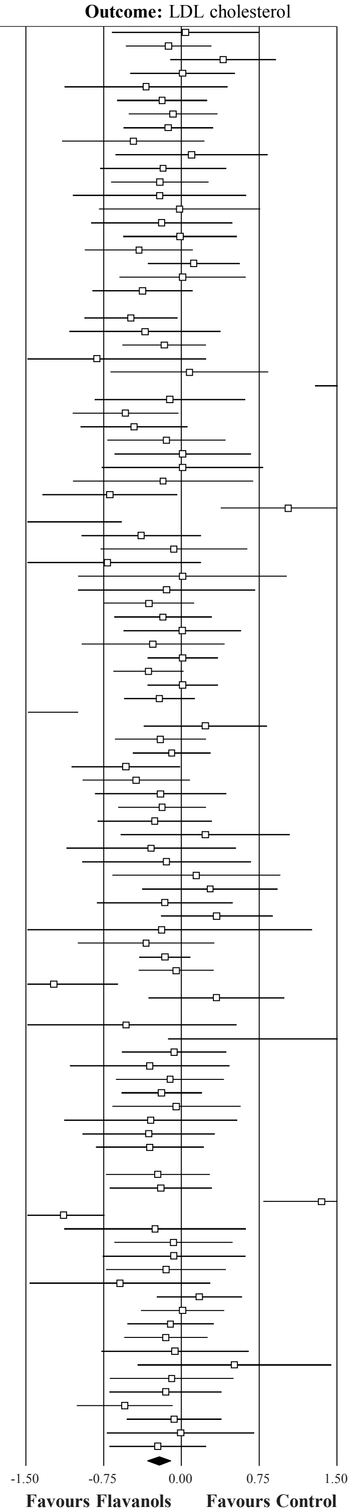

Test for overall effect Z=-4.171; *P*=0.000; F=2928, C=2798

Heterogeneity Tau^2=0.229; Q=408.0; df=104(*P*=0.000); I^2=74.5
