# Supplementary material for: A Systematic Review and Meta-Analysis of the Effects of Flavanol-Containing Tea, Cocoa and Apple Products on Body Composition and Blood Lipids: Exploring the Factors Responsible for Variability in Their Efficacy
Source: Nutrients. 2017 Jul 13;9(7):746. doi: 10.3390/nu9070746 (PMC5537860; doi:10.3390/nu9070746)
Supplement: Supplementary file 1 [file nutrients-09-00746-s001.zip › Figure S5.pdf]

Figure S5.- Forest plot of the meta-analysis evaluating the effects of a prolonged supplementation with flavanols-containing tea, cocoa or apple products on human blood levels of HDL cholesterol. A total of 112 studies (displayed in alphabetical order) were analysed. Pooled results are shown at the bottom using a random-effects model. SDM: standardized difference in means, SE: standard error, 95% CI: lower and upper confidence limits for the average SDM, RW: relative weight.

First author & year	SDM	SE	95% CI		RW
Akazome 2010	0.002	0.365	-0.714	0.718	0.82
Akazome 2010	0.059	0.215	-0.361	0.480	1.10
Al-Faris 2008	0.391	0.263	-0.124	0.906	1.01
Al-Faris 2008	0.000	0.260	-0.510	0.510	1.01
Baba 2007a	2.532	0.537	1.479	3.584	0.57
Baba 2007b	0.265	0.225	-0.175	0.705	1.08
Baba 2007b	0.123	0.224	-0.316	0.562	1.08
Baba 2007b	0.239	0.224	-0.201	0.679	1.08
Bahorun 2012	0.832	0.364	0.119	1.545	0.83
Bahorun 2012	1.067	0.394	0.295	1.838	0.77
Bajerska 2015	0.000	0.302	-0.592	0.592	0.94
Balzer 2008	0.298	0.314	-0.318	0.913	0.91
Barth 2012	-0.016	0.244	-0.494	0.463	1.04
Basu 2010	0.733	0.414	-0.077	1.544	0.74
Basu 2011	-0.675	0.440	-1.537	0.188	0.70
Basu 2011	-0.144	0.401	-0.929	0.642	0.76
Belcaro 2013	3.089	0.423	2.259	3.918	0.73
Belcaro 2013	2.062	0.347	1.383	2.741	0.86
Bingham 1997	0.023	0.283	-0.532	0.577	0.97
Bogdanski 2012	0.392	0.270	-0.137	0.921	1.00
Bøhn 2014	0.032	0.228	-0.415	0.479	1.07
Bondia-Pons 2014	-0.208	0.317	-0.829	0.414	0.91
Brown 2009	-1.655	0.247	-2.139	-1.170	1.04
Brown 2011	0.000	0.246	-0.483	0.483	1.04
Chai 2012	1.460	0.226	1.018	1.903	1.08
Chen 2016	-0.192	0.228	-0.640	0.256	1.07
Coimbra 2006	0.181	0.372	-0.549	0.911	0.81
Crews 2008	-0.009	0.211	-0.422	0.404	1.11
Davies 2003	-0.258	0.520	-1.277	0.760	0.59
Davison 2008	0.000	0.392	-0.769	0.769	0.78
Davison 2008	0.222	0.419	-0.599	1.042	0.73
de Jesus Romero-Prado 2015	0.088	0.225	-0.354	0.529	1.08
De la Torre 2014	-0.211	0.374	-0.945	0.523	0.81
Desideri 2012	-0.027	0.260	-0.537	0.483	1.01
Desideri 2012	0.049	0.263	-0.466	0.564	1.01
Diepvens 2006	-0.032	0.296	-0.613	0.549	0.95
Dower 2015	-0.500	0.343	-1.173	0.173	0.86
Duffy 2001	0.000	0.400	-0.785	0.785	0.76
Erba 2005	0.103	0.448	-0.774	0.980	0.69
Farouque 2006	0.324	0.327	-0.316	0.965	0.89
Flammer 2012	0.385	0.319	-0.241	1.010	0.90
Frank 2009	0.148	0.349	-0.535	0.832	0.85
Fu 2011	0.555	0.456	-0.338	1.448	0.68
Fujita 2008	0.000	0.292	-0.573	0.573	0.95
Fukino 2008	-0.096	0.365	-0.812	0.620	0.82
Grassi 2005a	0.000	0.447	-0.877	0.877	0.69
Grassi 2005b	0.333	0.521	-0.688	1.355	0.59
Hodgson 2002	0.079	0.437	-0.778	0.936	0.71
Hodgson 2013	0.026	0.230	-0.424	0.476	1.07
Hsu 2006	0.007	0.577	-1.125	1.139	0.52
Hsu 2008	0.187	0.227	-0.258	0.633	1.08
Hsu 2011	0.073	0.243	-0.403	0.549	1.05
Ibero-Baraibar 2014	-0.629	0.299	-1.215	-0.043	0.94
Inami 2007	0.029	0.354	-0.666	0.723	0.84
Ishikawa 1997	0.397	0.447	-0.480	1.273	0.69
Kajimoto 2005	0.016	0.176	-0.329	0.360	1.17
Kajimoto 2005	0.072	0.176	-0.273	0.417	1.17
Kajimoto 2005	0.062	0.175	-0.282	0.406	1.17
Kajimoto 2003	0.028	0.258	-0.479	0.534	1.02
Koutelidakis 2014	0.014	0.306	-0.586	0.613	0.93
Liu 2014	0.366	0.230	-0.084	0.817	1.07
Maki 2009	0.046	0.194	-0.334	0.425	1.14
Mastroiacovo 2015	0.101	0.265	-0.418	0.621	1.01
Mastroiacovo 2015	0.068	0.265	-0.452	0.587	1.01
Matsuyama 2008	0.196	0.325	-0.442	0.834	0.89
Mellor 2010	0.613	0.591	-0.544	1.771	0.51
Mielgo-Ayuso 2014	-0.260	0.221	-0.692	0.172	1.09
Miyazaki 2013	0.051	0.283	-0.503	0.606	0.97
Monagas 2009	0.674	0.317	0.052	1.295	0.91
Mozaffari-Khosravi 2009	0.145	0.275	-0.394	0.684	0.99
Muniyappa 2008	0.000	0.742	-1.454	1.454	0.37
Nagao 2005	0.034	0.338	-0.629	0.696	0.87
Nagao 2007	-0.089	0.129	-0.342	0.165	1.24
Nickols-Richardson 2014	-8.841	1.611	-11.999	-5.683	0.10
Njike 2011	1.395	0.357	0.695	2.095	0.84
Osakabe 2004	-0.246	0.710	-1.637	1.145	0.40
Osakabe 2004	-1.123	0.761	-2.615	0.368	0.36
Osakabe 2004	0.405	0.714	-0.995	1.806	0.39
Parsaeyan 2014	-0.516	0.203	-0.914	-0.117	1.12
Pierin Batista 2009	-0.177	0.349	-0.862	0.507	0.85
Ravn-Haren 2013	-0.130	0.419	-0.952	0.693	0.73
Rostami 2015	0.040	0.259	-0.467	0.548	1.02
Rull 2015	0.423	0.397	-0.354	1.200	0.77
Ryu 2006	0.157	0.270	-0.372	0.687	1.00
Sansone 2015	0.025	0.200	-0.367	0.417	1.13
Shiina 2009	0.009	0.320	-0.619	0.637	0.90
Shimada 2004	0.000	0.426	-0.836	0.836	0.72
Sone 2011	0.108	0.280	-0.441	0.658	0.98
Stendell-Hollis 2010	0.250	0.327	-0.390	0.890	0.89
Suliburska 2012	0.423	0.270	-0.107	0.953	1.00
Taubert 2007	-0.216	0.314	-0.832	0.399	0.91
Toolsee 2013	-0.068	0.259	-0.575	0.440	1.02
Toolsee 2013	-0.089	0.252	-0.584	0.405	1.03
Troup 2015	4.157	0.471	3.234	5.081	0.66
Tsai 2009	1.020	0.199	0.629	1.410	1.13
Tzounis 2011	-0.104	0.448	-0.981	0.773	0.69
Vafa 2011	-0.103	0.295	-0.681	0.475	0.95
Velliquette 2015	-0.154	0.355	-0.849	0.542	0.84
Vieira Senger 2012	0.000	0.299	-0.586	0.586	0.94
Vlachopoulos 2005	0.198	0.427	-0.640	1.036	0.72
Wang 2000	0.014	0.211	-0.400	0.428	1.11
Wang 2000	-0.000	0.209	-0.410	0.410	1.11
Wang 2010	-0.054	0.216	-0.477	0.368	1.10
Wang 2010	0.131	0.209	-0.279	0.541	1.11
West 2014	0.333	0.368	-0.387	1.054	0.82
Westphal 2011	1.000	0.500	0.020	1.980	0.62
Widlansky 2007	0.181	0.309	-0.425	0.787	0.92
Widmer 2013	0.074	0.278	-0.471	0.620	0.98
Wu 2012	0.033	0.236	-0.429	0.495	1.06
Wu 2012	-0.122	0.238	-0.587	0.344	1.06
Yang 2012	-0.722	0.379	-1.464	0.021	0.80
Yang 2014	-0.177	0.240	-0.647	0.292	1.05
**Intervention effect**	**0.152**	**0.053**	**0.047**	**0.256**	

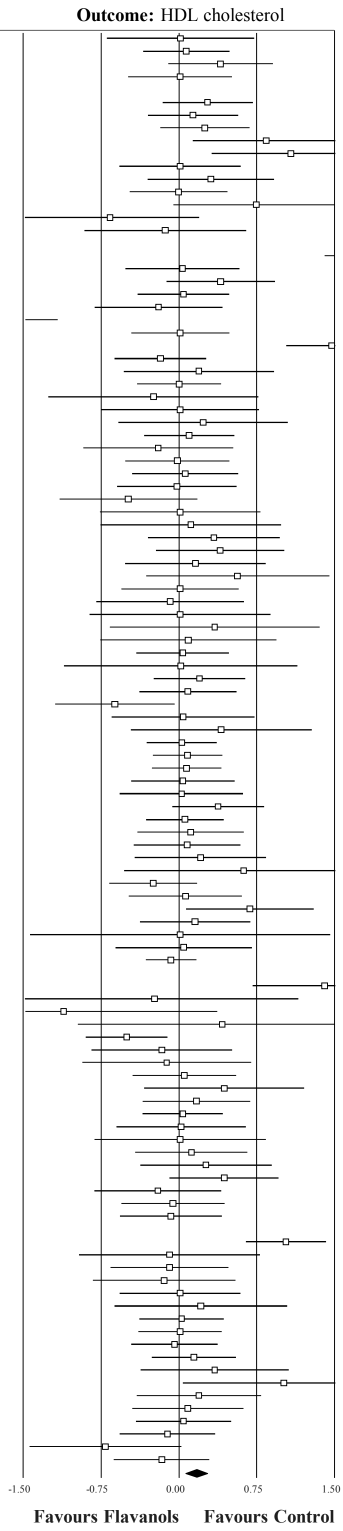

Test for overall effect Z=2.836; *P*=0.005; F=3023, C=2905

Heterogeneity Tau^2=0.214; Q=408.0; df=111(*P*=0.000); I^2=72.8
