# Supplementary material for: A Systematic Review and Meta-Analysis of the Effects of Flavanol-Containing Tea, Cocoa and Apple Products on Body Composition and Blood Lipids: Exploring the Factors Responsible for Variability in Their Efficacy
Source: Nutrients. 2017 Jul 13;9(7):746. doi: 10.3390/nu9070746 (PMC5537860; doi:10.3390/nu9070746)
Supplement: Supplementary file 1 [file nutrients-09-00746-s001.zip › Figure S6.pdf]

Figure S6.- Forest plot of the meta-analysis evaluating the effects of a prolonged supplementation with flavanols-containing tea, cocoa or apple products on human blood levels of triglycerides (TAGs). A total of 120 studies (displayed in alphabetical order) were analysed. Pooled results are shown at the bottom using a random-effects model. SDM: standardized difference in means, SE: standard error, 95% CI: lower and upper confidence limits for the average SDM, RW: relative weight.

First author & year	SDM	SE	95% CI		RW
Akazome 2010	-0.000	0.365	-0.717	0.716	0.77
Akazome 2010	-0.080	0.215	-0.501	0.340	1.04
Al-Faris 2008	0.000	0.260	-0.510	0.510	0.96
Al-Faris 2008	0.333	0.262	-0.181	0.847	0.95
Baba 2007a	-1.041	0.427	-1.877	-0.205	0.68
Baba 2007b	-0.075	0.224	-0.514	0.363	1.02
Baba 2007b	-0.126	0.224	-0.564	0.313	1.02
Baba 2007b	-0.110	0.224	-0.549	0.328	1.02
Bahorun 2012	1.029	0.371	0.303	1.756	0.76
Bahorun 2012	-0.495	0.380	-1.240	0.250	0.75
Bajerska 2015	0.000	0.302	-0.592	0.592	0.88
Balzer 2008	-0.299	0.314	-0.915	0.317	0.86
Barth 2012	0.206	0.245	-0.274	0.686	0.99
Basu 2011	0.000	0.428	-0.839	0.839	0.68
Basu 2011	-0.135	0.401	-0.921	0.650	0.72
Belcaro 2013	-1.328	0.223	-1.765	-0.890	1.02
Bingham 1997	0.016	0.283	-0.538	0.570	0.92
Bogdanski 2012	-0.600	0.273	-1.135	-0.065	0.93
Bøhn 2014	-0.018	0.228	-0.465	0.429	1.02
Bondia-Pons 2014	-0.493	0.321	-1.122	0.136	0.85
Brown 2009	2.282	0.274	1.745	2.820	0.93
Brown 2011	0.147	0.248	-0.340	0.634	0.98
Chen 2016	-0.294	0.229	-0.743	0.155	1.01
Coimbra 2006	0.079	0.372	-0.650	0.808	0.76
Crews 2008	0.183	0.211	-0.231	0.597	1.05
Davies 2003	-0.261	0.537	-1.314	0.791	0.53
Davison 2008	-0.716	0.405	-1.509	0.077	0.71
Davison 2008	-0.237	0.419	-1.058	0.584	0.69
de Jesus Romero-Prado 2015	-0.822	0.235	-1.282	-0.362	1.00
De la Torre 2014	-0.043	0.373	-0.775	0.689	0.76
Desideri 2012	-0.114	0.261	-0.625	0.397	0.96
Desideri 2012	-0.287	0.264	-0.805	0.230	0.95
Diepvens 2006	-0.115	0.295	-0.694	0.464	0.89
Dower 2015	-0.200	0.339	-0.865	0.465	0.82
Duffy 2001	0.073	0.400	-0.711	0.858	0.72
Erba 2005	0.003	0.447	-0.874	0.879	0.65
Esser 2014	0.000	0.372	-0.728	0.728	0.76
Esser 2014	-0.100	0.313	-0.713	0.513	0.86
Farouque 2006	0.185	0.325	-0.453	0.822	0.84
Flammer 2012	-0.222	0.317	-0.844	0.400	0.86
Frank 2009	-0.230	0.349	-0.915	0.455	0.80
Freese 1999	0.000	0.447	-0.877	0.877	0.65
Fu 2011	-0.287	0.450	-1.168	0.594	0.64
Fujita 2008	-0.167	0.293	-0.741	0.407	0.90
Fukino 2008	0.036	0.366	-0.681	0.753	0.77
Grassi 2005a	-0.500	0.454	-1.390	0.390	0.64
Grassi 2005b	-0.286	0.520	-1.305	0.734	0.55
Hodgson 2013	0.203	0.230	-0.248	0.654	1.01
Hodgson 2002	0.000	0.437	-0.856	0.856	0.66
Hsu 2011	0.101	0.243	-0.375	0.577	0.99
Hsu 2008	0.440	0.229	-0.010	0.890	1.01
Hsu 2006	-0.393	0.583	-1.535	0.750	0.48
Hursel 2009	0.000	0.316	-0.620	0.620	0.86
Ibero-Baraibar 2014	-0.181	0.292	-0.755	0.392	0.90
Inami 2007	-0.290	0.356	-0.987	0.406	0.79
Ishikawa 1997	0.348	0.446	-0.527	1.223	0.65
Kajimoto 2003	0.145	0.259	-0.362	0.652	0.96
Koutelidakis 2014	-0.165	0.306	-0.765	0.435	0.88
Kovacs 2004	0.251	0.198	-0.136	0.639	1.07
Lettieri-Barbato 2012	-0.161	0.520	-1.180	0.859	0.55
Liu 2014	-0.383	0.230	-0.834	0.068	1.01
Maki 2009	-0.447	0.196	-0.831	-0.063	1.07
Mastroiacovo 2015	-0.031	0.265	-0.551	0.488	0.95
Mastroiacovo 2015	-0.201	0.266	-0.721	0.320	0.95
Matsuyama 2008	-0.130	0.325	-0.767	0.507	0.84
Mellor 2010	-0.096	0.408	-0.897	0.704	0.71
Mielgo-Ayuso 2014	-0.246	0.221	-0.678	0.186	1.03
Miyazaki 2013	-0.507	0.287	-1.071	0.056	0.91
Monagas 2009	0.045	0.309	-0.560	0.650	0.87
Mousavi 2013	0.117	0.334	-0.537	0.772	0.83
Mousavi 2013	-0.497	0.341	-1.165	0.172	0.81
Mozaffari-Khosravi 2009	0.448	0.278	-0.098	0.993	0.93
Muniyappa 2008	0.000	0.316	-0.620	0.620	0.86
Nagao 2005	0.043	0.222	-0.392	0.478	1.03
Nagao 2007	-0.020	0.129	-0.273	0.234	1.17
Nagao 2009	-0.257	0.307	-0.858	0.345	0.87
Nickols-Richardson 2014	0.398	0.290	-0.170	0.967	0.90
Njike 2011	-0.082	0.320	-0.710	0.547	0.85
Osakabe 2004	0.000	0.707	-1.386	1.386	0.37
Osakabe 2004	-0.110	0.708	-1.497	1.277	0.37
Osakabe 2004	-0.056	0.707	-1.442	1.330	0.37
Parsaeyan 2014	-2.773	0.280	-3.322	-2.224	0.92
Pierin Batista 2009	0.093	0.349	-0.590	0.776	0.80
Ravn-Haren 2013	-0.229	0.419	-1.049	0.592	0.69
Ravn-Haren 2013	0.166	0.418	-0.653	0.986	0.69
Ravn-Haren 2013	0.096	0.418	-0.722	0.915	0.69
Ravn-Haren 2013	-0.124	0.418	-0.943	0.695	0.69
Rostami 2015	-0.221	0.260	-0.730	0.287	0.96
Ryu 2006	0.089	0.270	-0.440	0.618	0.94
Sansone 2015	0.070	0.200	-0.322	0.462	1.06
Chai 2012	-2.135	0.268	-2.661	-1.610	0.94
Shiina 2009	0.312	0.322	-0.319	0.944	0.85
Shimada 2004	-0.261	0.428	-1.101	0.578	0.68
Sone 2011	0.393	0.283	-0.161	0.947	0.92
Stendell-Hollis 2010	0.000	0.326	-0.638	0.638	0.84
Taubert 2007	-0.100	0.316	-0.719	0.520	0.86
Tinahones 2008	-1.231	0.583	-2.373	-0.088	0.48
Toolsee 2013	-0.360	0.261	-0.871	0.151	0.96
Toolsee 2013	0.238	0.253	-0.258	0.734	0.97
Troup 2015	-1.669	0.308	-2.272	-1.066	0.87
Tzounis 2011	-2.357	0.582	-3.498	-1.216	0.48
Unno 2005	-0.274	0.674	-1.596	1.048	0.40
Unno 2005	-0.510	0.683	-1.848	0.828	0.39
Vafa 2011	0.620	0.302	0.029	1.212	0.88
Velliquette 2015	-0.009	0.354	-0.704	0.685	0.79
Vieira Senger 2012	0.243	0.300	-0.345	0.830	0.89
Villano 2010	1.156	0.566	0.047	2.265	0.50
Vlachopoulos 2005	0.757	0.452	-0.129	1.644	0.64
Wang 2010	0.004	0.216	-0.419	0.426	1.04
Wang 2010	-0.042	0.209	-0.451	0.368	1.05
Wang 2000	-0.163	0.212	-0.578	0.252	1.04
Wang 2000	-0.010	0.209	-0.419	0.400	1.05
West 2014	0.191	0.366	-0.526	0.908	0.77
Westphal 2011	0.011	0.472	-0.914	0.936	0.61
Widlansky 2007	-0.162	0.309	-0.768	0.443	0.87
Widmer 2013	0.108	0.278	-0.438	0.653	0.93
Wu 2012	-0.171	0.236	-0.634	0.292	1.00
Wu 2012	0.421	0.240	-0.049	0.891	0.99
Yang 2012	-0.245	0.367	-0.964	0.474	0.77
Yang 2014	0.019	0.239	-0.449	0.488	1.00
**Intervention effect**	**-0.114**	**0.051**	**-0.215**	**-0.013**	

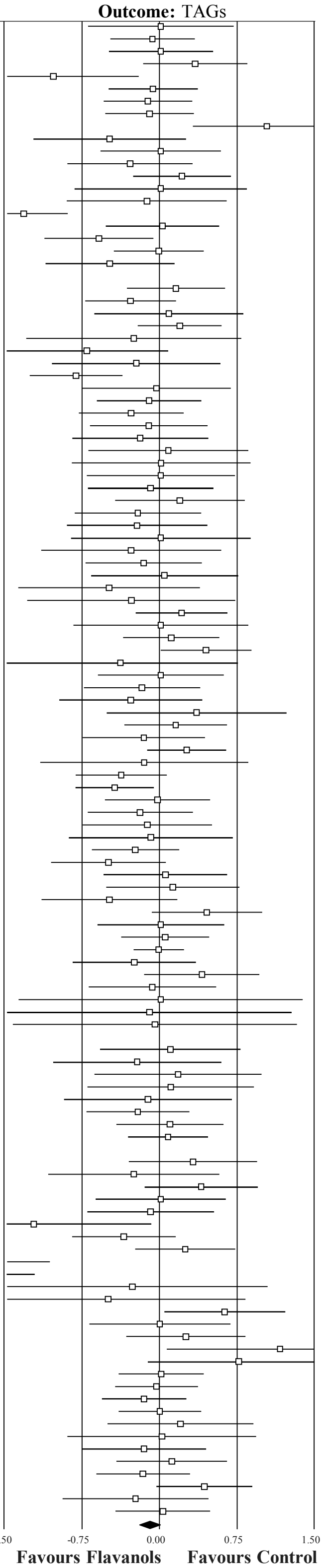

Test for overall effect Z=-2.213; P=0.027; F=3023, C=2908

Heterogeneity Tau^2=0.209; Q=407.7; df=119(P=0.000); I^2=70.8
