# Supplementary material for: A Systematic Review and Meta-Analysis of the Effects of Flavanol-Containing Tea, Cocoa and Apple Products on Body Composition and Blood Lipids: Exploring the Factors Responsible for Variability in Their Efficacy
Source: Nutrients. 2017 Jul 13;9(7):746. doi: 10.3390/nu9070746 (PMC5537860; doi:10.3390/nu9070746)

Figure S10.- Funnel plot and Eager statistics (intercept and 2-tailed *P*-value) of the meta-analysis evaluating the effects of a prolonged supplementation with flavanols-containing tea, cocoa or apple products on human blood levels of LDL cholesterol.

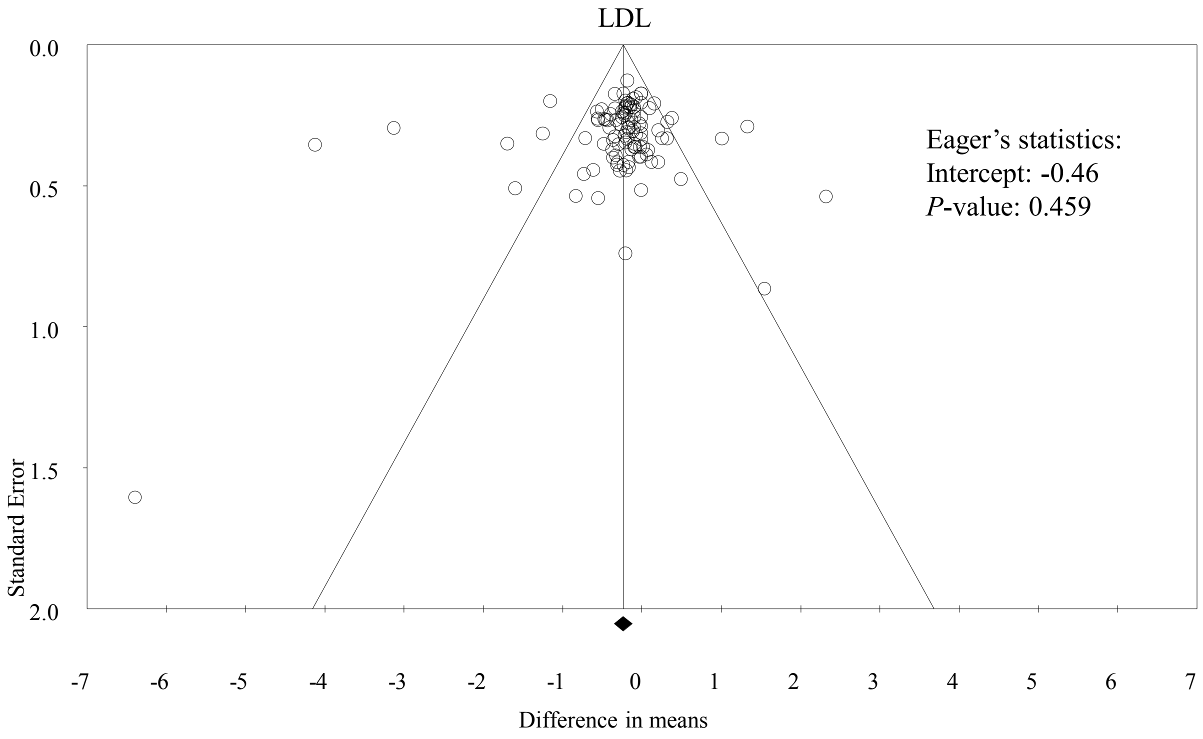

Supplement: Supplementary file 1 [file nutrients-09-00746-s001.zip › Figure S10.pdf]
