# Supplementary material for: A Systematic Review and Meta-Analysis of the Effects of Flavanol-Containing Tea, Cocoa and Apple Products on Body Composition and Blood Lipids: Exploring the Factors Responsible for Variability in Their Efficacy
Source: Nutrients. 2017 Jul 13;9(7):746. doi: 10.3390/nu9070746 (PMC5537860; doi:10.3390/nu9070746)
Supplement: Supplementary file 1 [file nutrients-09-00746-s001.zip › Table S1.pdf]

Table S1.- Mean content (mg/100 g fresh weight, f.w., or mg/100 mL) in flavonoids of green and black tea infusions, cocoa powder and whole apple illustrative of the composition of the three main sources of flavanols examined in this study: tea, cocoa and apple (data are based on the Phenol-Explorer database).

Flavonoids		Green Tea Infusion (mg/100 mL)	Black Tea Infusion (mg/100 mL)	Cocoa Powder (mg/100 g f.w.)	Apple [Cider] peeled (mg/100 g f.w.)
Flavanols	(+)-Catechin	0.70	2.45	107.75	5.56
	(+)-Catechin 3-O-gallate	-	4.74	-	-
	(+)-Gallocatechin	2.26	14.01	-	-
	(+)-Gallocatechin 3-O-gallate	0.47	0.67	-	-
	(-)-Epicatechin	7.93	3.94	158.30	28.67
	(-)-Epicatechin-(2a-7)(4a-8)-epicatechin 3-O-galactoside	-	-	5.00	-
	(-)-Epicatechin 3-O-gallate	7.50	7.34	-	-
	(-)-Epigallocatechin	19.68	7.19	-	-
	(-)-Epigallocatechin 3-O-gallate	27.16	9.12	-	-
	Cinnamtannin A2	-	-	33.17	-
	Procyanidins (dimers, trimers)	5.48	11.38	207.4	26.02
	TOTAL	71.18	60.84	511.62	60.25
Flavonols	Kaempferol	-	0.00636	-	-
	Kaempferol 3-O-galactoside	0.42	0.35	-	-
	Kaempferol 3-O-glucoside	1.29	0.90	-	-
	Kaempferol 3-O-glucosyl-rhamnosyl-galactoside	-	0.28	-	-
	Kaempferol 3-O-glucosyl-rhamnosyl-glucoside	-	0.67	-	-
	Kaempferol 3-O-rutinoside	0.95	1.32	-	-
	Quercetin	-	0.00364	-	-
	Quercetin 3-O-arabinoside	-	0.02	-	-
	Quercetin 3-O-galactoside	0.97	0.73	-	0.00829
	Quercetin 3-O-glucoside	-	1.31	-	0.06
	Quercetin 3-O-glucosyl-rhamnosyl-galactoside	-	0.67	-	-
	Quercetin 3-O-glucosyl-rhamnosyl-glucoside	-	1.10	-	-
	Quercetin 3-O-rhamnoside	-	0.06	-	0.22
	Quercetin 3-O-rhamnosyl-galactoside	0.20	0.27	-	-
	Quercetin 3-O-rutinoside	1.46	1.62	-	-
	TOTAL	5.29	9.31	-	0.29
Dihydro chalcones	Phloretin 2'-O-xylosyl-glucoside	-	-	-	3.28
	Phloridzin	-	-	-	2.50
	TOTAL	-	-	-	5.78
