# Supplementary material for: A Systematic Review and Meta-Analysis of the Effects of Flavanol-Containing Tea, Cocoa and Apple Products on Body Composition and Blood Lipids: Exploring the Factors Responsible for Variability in Their Efficacy
Source: Nutrients. 2017 Jul 13;9(7):746. doi: 10.3390/nu9070746 (PMC5537860; doi:10.3390/nu9070746)
Supplement: Supplementary file 1 [file nutrients-09-00746-s001.zip › Table S2.pdf]

Table S2.- Results of the meta-regression of the changes (SDM) in BMI, WC and blood lipids levels *vs.* duration of the supplementation with the flavanol-containing tea, cocoa or apple products.

	BMI	WC	Total-cholesterol	LDL-cholesterol	HDL-cholesterol	TAGs
Slope	-0.0007	-0.0028	-0.0064	-0.0066	0.0038	-0.0044
SE	0.0008	0.0013	0.0012	0.0014	0.0010	0.0009
95% CI	-0.0023, 0.0008	-0.0054, -0.0002	-0.0088, -0.0040	-0.0093, -0.0039	0.0018, 0.0058	-0.0061, -0.0026
2-sided *P*-value	0.350	0.032	<0.001	<0.001	<0.001	<0.001
Number of studies	60	68	154	141	145	166

SDM: Standard difference in means; BMI: Body Mass Index; WC: Waist Circumference; LDL: Low density Lipoprotein; HDL: High Density Lipoprotein; TAGs: Triacylglycerides; Slope: Regression Coefficient; SE: Standard Error; 95% CI: lower and upper confidence limits; *P*-value < 0.05 was considered significant.
