# Supplementary material for: A Systematic Review and Meta-Analysis of the Effects of Flavanol-Containing Tea, Cocoa and Apple Products on Body Composition and Blood Lipids: Exploring the Factors Responsible for Variability in Their Efficacy
Source: Nutrients. 2017 Jul 13;9(7):746. doi: 10.3390/nu9070746 (PMC5537860; doi:10.3390/nu9070746)
Supplement: Supplementary file 1 [file nutrients-09-00746-s001.zip › Table S3.pdf]

Table S3.- Summary of recent meta-analysis looking at the effects of flavanol-containing tea or cocoa products on anthropometric measurements and blood lipids associated with the development of metabolic disorders.

Product	Maximum number of studies	Maximum number of participants	BMI	WC (cm)	Total cholesterol (mmol/L)	LDL (mmol/L)	HDL (mmol/L)	TAGs (mmol/L)	References
Tea products meta-analyses									
Various types of tea or tea extracts	13	665	-0.15 (NS)	-1.01 (NS)	-0.05 (NS)	0.07 (NS)	0.01 (NS)	-0.11 (NS)	[136]
Black tea	10	411	NR	NR	-0.05 (NS)	-0.12 (S)	0.03 (NS)	NR	[137]
Various types of tea or tea extracts	4	302	-0.72 (S)	-1.53 (NS)	NR	NR	NR	NR	[138]
Black tea extracts, leafs	12	-	NR	NR	NE	-0.12 (NS)	NE	NR	[139]
Green tea extracts or drinks	20	1536	NR	NR	-0.13 (S)	-0.19 (S)	-0.01 (NS)	-0.02 (NS)	[140]
Green tea extracts or drinks	5	301	-0.31 (NS)	0.08 (NS)	NR	NR	NR	NR	[141]
Green tea extracts or drinks	11	927	-0.06 (NS)	NR	-0.15 (S)	-0.16 (S)	0.01 (NS)	0.10 (NS)	[142]
Green and black tea, extracts	8	821	NR	NR	NR	-0.48 (S)	0.00 (NS)	-0.06 (NS)	[143]
Green tea extracts or drinks	20	1415	NR	NR	-0.14 (S)	-0.14 (S)	0.01 (NS)	0.03 (NS)	[144]
Green tea extracts or drinks	14	1136	NR	NR	-0.19 (S)	-0.06 (S)	0.25 (NS)	NR	[145]
Green tea extracts or drinks (with caffeine)	15	1243	-0.55 (S)	-1.93 (S)	NR	NR	NR	NR	[146]
Cocoa products meta-analyses									
Dark chocolate/cocoa products	10	320	NR	NR	-0.16 (S)	-0.15 (S)	0.02 (NS)	-0.06 (NS)	[147]
Cocoa, chocolate bars or beverages	20	1131	NR	NR	-0.07 (MS)	-0.26 (MS)	0.06 (S)	-0.10 (S)	[148]
Chocolate and cocoa flavanols	21	986	NR	NR	-0.04 (NS)	-0.07 (MS)	0.03 (MS)	-0.05 (S)	[149]

BMI: Body Mass Index; WC: Waist Circumference; LDL: Low density Lipoprotein; HDL: High Density Lipoprotein; TAGs: Triacylglycerides; NR: Not reported; NE: No effect; S: Significant (P<0.05, dark shaded); MS: Marginally significant (P<0.1 and ≥ 0.05); NS: Not significant.
