# Supplementary material for: A Systematic Review and Meta-Analysis of the Effects of Flavanol-Containing Tea, Cocoa and Apple Products on Body Composition and Blood Lipids: Exploring the Factors Responsible for Variability in Their Efficacy
Source: Nutrients. 2017 Jul 13;9(7):746. doi: 10.3390/nu9070746 (PMC5537860; doi:10.3390/nu9070746)
Supplement: Supplementary file 1 [file nutrients-09-00746-s001.zip › Table S4.pdf]

Table S4.- Overall effect size estimations (DM) for the impact of flavanols containing products on BMI, WC and blood lipids levels.

	n	N_T	N_S	N_C	Overall effect size (DM)	95% CI	*P*-value
BMI (kg/m^2)	67	3726	1913	1813	-0.146	-0.252, -0.041	0.006
WC (cm)	46	2875	1478	1397	-1.671	-2.432, -0.909	<0.001
Total-cholesterol (mmol/L)	103	5190	2671	2519	-0.134	-0.217, -0.050	0.002
LDL-cholesterol (mmol/L)	99	5268	2697	2571	-0.170	-0.251, -0.089	<0.001
HDL-cholesterol (mmol/L)	103	5328	2722	2606	0.032	-0.006, 0.071	0.099
TAGs (mmol/L)	114	5561	2840	2721	-0.031	-0.065, 0.004	0.083

DM: Difference in means; BMI: Body Mass Index; WC: Waist Circumference; LDL: Low density Lipoprotein; HDL: High Density Lipoprotein; TAGs: Triacylglycerides; n: total number of studies included in the analysis; N_T: number of total participants; N_S: number of participants in the supplemented group; N_C: number of participants in the control group; DM: Difference in Means (absolute values); 95% CI: lower and upper confidence limits; *P*-value < 0.05 was considered significant; *P*-value < 0.1 and ≥ 0.05 was considered marginally significant.
